# Metagenomics: An Approach for Unraveling the Community Structure and Functional Potential of Activated Sludge of a Common Effluent Treatment Plant

**DOI:** 10.3389/fmicb.2022.933373

**Published:** 2022-07-18

**Authors:** Gunjan Vasudeva, Harpreet Singh, Sakshi Paliwal, Anil Kumar Pinnaka

**Affiliations:** MTCC-Microbial Type Culture Collection and Gene Bank, CSIR-Institute of Microbial Technology, Chandigarh, India

**Keywords:** metagenomics, antimicrobial resistance, effluent treatment plant (ETP), metagenomic binning, functional annotation

## Abstract

The common effluent treatment plant (CETP) located at Baddi treats the industrial effluent from various industries, leading to the pooling of a diverse range of substrates and metabolites. The nutrient loading and its availability decide the balance of the microbial community and its diversity. The samples thus collected from the activated sludge (BS14) of CETP and Sirsa river (SR1) from the vicinity of CETP effluent discharge were processed for the whole metagenome analysis to reveal the microbial community and its functional potential. The taxonomic classification of the BS14 sample showed the dominance of the bacterial community with 96% of abundance, whereas the SR1 was populated by eukaryotes representing 50.4% of the community of SR1. The bacterial community of SR1 was constituted of 47.2%. The functional analysis of BS14 and SR1 with GhostKOALA against the KEGG database assigned 43.7% and 27.8% of the open reading frames (ORFs) with functions. It revealed the xenobiotic degradation modules with complete pathways along with resistance against the beta-lactams. The analysis with the comprehensive antibiotic resistance database (CARD) revealed 33 and 32 unique types of antimicrobial resistance in BS14 and SR1, respectively. Both the samples were dominated by the beta-lactam resistance genes. The carbohydrate-active enzyme (CAZy) database assigned a total of 6,611 and 2,941 active enzymes to BS14 and SR1, respectively. In contrast, the glycosyl hydrolases (GH) and glycosyltransferases (GT) class of enzymes were found to be abundant in both the samples as compared with polysaccharide lyases (PL), auxiliary activities (AA), carbohydrate esterases (CE), and carbohydrate-binding module (CBM).

## Introduction

Multi-omics, also known as integrated omics, is a modern field of biology that combines more than one biology-based omics data to explore the structure and interactions of the complex biological system at the individual and community level (Krassowski et al., 2020). The different applications of omics studies include genomics (DNA-based), metagenomics (community DNA), transcriptomics (all RNA-based), epigenomics, pan genomics, proteomics (protein-based), and metabolomics (metabolites content) (Bersanelli et al., 2016; Bock et al., 2016; Vilanova and Porcar, 2016). The different approaches to omics and the identification of biological markers obtained from DNA, RNA, proteins, and metabolites also helped to understand the various biological processes, health diseases, microbiology, and other environment-related processes (Marvasi et al., 2021).

The multi-omics profiling of unique niches for unraveling the microbial community and their metabolite composition involves a variety of high-throughput data, including 16S, shotgun metagenomics, metatranscriptomics, and metabolomics (Shaffer et al., 2021). The microbial communities are a crucial part of all lives on earth, and they maintain all the biogeochemical cycles on the earth by cycling the element between the lithosphere, atmosphere, hydrosphere, and biosphere (Madsen, 2011; Griggs et al., 2013). The advancement of next-generation sequencing (NGS) and omics technologies has generated high throughput data and analysis platforms to study the structure of microbial communities on-site, bypassing the need for pure culture isolation (Unamba et al., 2015; Costessi et al., 2018; Ben Khedher et al., 2022).

Wastewater treatment plants (WWTPs) are specialized systems that collects and treats wastewater for downstream usage to improve the quality of human and aquatic life (Waldrop, 2021). The increased urbanization and industrialization pose a severe risk to all living forms by increasing the accumulation of toxic pollutants (McMichael, 2000; Satterthwaite et al., 2010) and can also disseminate a load of various pathogens and antibiotic resistance genes, thus should be subjected for biological and chemical treatment (Mukherjee et al., 2021; Nguyen et al., 2021). Common effluent treatment plants (CETPs) offer a combined system to treat the wastewater collected from various small and medium-scale local industries (Padalkar and Kumar, 2018). ~193 CETPs are established in India for primary to secondary treatment to increase water reusability for nonpotable purposes (Ali et al., 2021). The secondary treatment of wastewater involves the application of activated sludge microbiome (bacteria, archaea, protists, and fungi) to remove the dissolved and suspended organic matter, measurable by biological oxygen demand by 90%, both aerobically and anaerobically (Narayanan and Narayan, 2019).

The primary aim of CETPs and ETPs is to remove toxic, hazardous compounds before being discharged into the environment, primarily aquatic systems. Along with hazardous compounds, the CETP incoming water has a high concentration of pharmaceutical products, antibiotics, and heavy metals (Hubeny et al., 2021; Zieliński et al., 2021). The high presence of antibiotics in wastewater also increases the selective pressure to exchange antibiotic resistance genes among the microbes (Kraemer et al., 2019). Microbiome profiling of wastewater, activated sludge of effluent treatment plants, and receiving aquatic environment have already been reported in many studies through culture-dependent, quantitative PCR (qPCR), and culture-independent methods, e.g., 16S and shotgun metagenomics (Chu et al., 2018). Due to the limitation of the culturability of microbes, the whole spectrum of microbial diversity and their functions cannot be captured; however, using shotgun metagenomics, it is possible to untap the hidden microbial community and their interactions in any given environmental sample (Handelsman, 2004; Bodor et al., 2020). Using shotgun metagenomics analysis, the influence of wastewater on the genetic composition of sediment microflora was observed in terms of the dissemination of antibiotic resistance gene mobile genetic elements into the receiving aquatic system (Matviichuk et al., 2022).

The primary objective of this study was microbiome analysis of activated sludge of CETP, Baddi, and its receiving freshwater Sirsa river (SR1). To fulfill our objective, the high throughput shotgun metagenomic sequencing data using the Illumina NextSeq500 platform was obtained. The taxonomic and functional profiling was performed for both samples to elucidate microbial community composition and study their metabolic capabilities. This study could help understand the similarities and differences between the microbial and functional potential of both sites and shed light on wastewater's influence on the microbial and genetic composition of its receiving freshwater.

## Materials and Methods

### Site Description and Sample Collection

Samples were collected from CETP established at Baddi Himachal Pradesh, India. Baddi-Barotiwala-Nalagarh (BBN) is one of the India's most extensive industrial belts and is the world's third-largest pharma hub. Baddi was declared a severely polluted area by Central Pollution Control Board in 2006 (Cluster, 2018). In this regard, the CETP was established in 2005–2006 in Kenduwal village (30.95°N 76.79°E) to treat the effluent load of 25 MLD (Million Liters per Day) from the various industries (e.g., textile, food, paper, detergent, pharmaceutical, and dye industries) in the BBN chain. Due to the massive variety of waste received from the BBN industrial belt, the CETP provides an artificially enriched environment for developing a highly efficient and dynamic microbial community.

The other sample collection was performed from the CETP water receiving fresh river SR1, Himachal Pradesh, Baddi. The SR1 arises in the Shivalik foothill of southern Himachal Pradesh and flows from Solan to the BBN area (29° 32'5.60 “N 75°01'44.33” E), and it meets the Sutlej River in Punjab. The various reports by the Centre of Pollution Control Board and other channels note that the extensive industrial waste is being dumped into the SR1 in the BBN area by CETP and other industries. We have collected the samples from the SR1 ~100 m away from the drainage site of CETP.

### DNA Extraction and Metagenomic Sequencing

The samples were collected in sterile containers, transferred to the laboratory as soon as possible, and processed further for high-molecular-weight metagenomic DNA isolation using the Meta-G-Nome^TM^ DNA Isolation Kit, Epicenter. The BS14 and SR1 samples were submitted for whole metagenomic sequencing to generate paired-end type reads (2 × 150) in NextSeq500 (Illumina).

### Read Processing

The quality of the raw reads was assessed through FastQC (version 0.11.9) (Andrews, 2010), and it was further processed with trimmomatic (version 0.39) (Bolger et al., 2014) to remove the adapters (Leading:33, Trailing:33, without the sliding-window feature). The raw reads were trimmed with the force trim modulo feature of BBDuk (BBMap version 38.93) (Bushnell, 2014) to correct the read length from 151 bps to 150 bps. The read quality was improved with the parameters—rtrim, qtrim = 30, minlen = 51 *via* BBDuk. FastQC analysis was used at every step to assess the sample-specific read processing.

### Metagenomic Assembly

The high-quality reads were assembled with a de-Bruijn graph-based assembler, i.e., metaSPAdes (version 3.15.3) (Nurk et al., 2017) at default parameters with a kmer value of 21, 33, 51, and 75 for both samples BS14 and SR-1. Using Bioawk (version 20110810), the contigs shorter than 1,000 bps were removed (Li, 2017).

### Taxonomic Profiling

The metagenome assembly of BS14 and SR-1 were analyzed using Kaiju (version 1.8.1) (Menzel et al., 2016) for the taxonomic classification of the whole metagenome. Kaiju generates the Burrows–Wheeler transform (bwt) and Ferragina-Manzini index (fmi) of the nr database (version 26-03-2022) for the alignment. It translates the contigs into six possible reading frames and aligns them against the fm-index of the nr database. The taxonomic classification of BS14 and SR1 was run at default parameters with greedy mode.

### Functional Profiling

The functional annotation of BS14 and SR1 assemblies was performed with the help of SqueezeMeta pipeline version 1.5.1 (Tamames and Puente-Sánchez, 2019). SqueezeMeta accepts the reads and does the automated processing and assembly followed by taxonomical and functional annotation of the genes. SqueezeMeta uses nr (Sayers et al., 2022), Pfam (Mistry et al., 2020), Clusters of Orthologous Genes (COG) (Tatusov et al., 2000), and Kyoto Encyclopedia of Genes and Genomes (KEGG) database for functional and taxonomic classification of the ORFs. The COG functions were manually extracted and annotated using DIAMOND (Buchfink et al., 2015) and visualized using ggplot2 (Wickham, 2016). The samples were also screened for the virulence factor through the virulence factor database (VFDB) analysis. The ORFs were aligned with VFDB to extract the relevant functions and visualized through ggplot2.

The amino acid sequences obtained from Prodigal (Hyatt et al., 2010) were further annotated using GhostKOALA (KEGG Orthology And Links Annotation), freely available on (https://www.kegg.jp/ghostkoala/) for assignment of KO numbers to infer functional categories and pathways for both metagenomes (Kanehisa et al., 2016).

### Antimicrobial Resistance Profiling

The antimicrobial resistance (AMR) profiling of both BS14 and SR1 assemblies was performed by mapping the predicted ORFs obtained from Prodigal against the CARD database (homologs) (Alcock et al., 2020) using blastx mode of DIAMOND with a minimum identity of 50, query coverage of 90, and minimum bit score of 50 in fast mode. The AMR profile of both metagenomes was linked to the genus level of taxonomy obtained by Kaiju by comparing the first common fields of two files to combine the contents with the help of awk. The total AMR content of both metagenomes BS14 and SR1 was also compared to determine the shared resistome.

### Carbohydrate Active Enzyme Profiling

The presence of carbohydrate-active enzymes in metagenome was determined by aligning the ORFs obtained by Prodigal to dbCAN database of carbohydrate-active enzyme (CAZy) annotation (Drula et al., 2022) using blastx mode of DIAMOND with 50% of minimum identity and 90% of query coverage with a minimum bit score of 50, in fast mode. Similarly, the polysaccharide utilization locus database (PULDB) was used to align against the ORFs of BS14 and SR1 (with the same parameters as used for the CAZy database) for the analysis of the polysaccharide metabolizing enzymes (Terrapon et al., 2018).

### Binning and Bin Refinement

The contigs of BS14 and SR1 were binned using maxbin2, metabat, and concoct at default parameters (Alneberg et al., 2014; Wu et al., 2016; Kang et al., 2019). The metagenome-assembled genomes (MAGs) were further refined using the DAS Tool (Sieber et al., 2018). The refined bins were checked for completeness, contamination, and taxonomy lineage using checkm version 1.1.3 (Parks et al., 2015) with its lineage-specific mode. The bins with completeness >90% and contamination <5% were selected for further analysis.

### Taxonomic Assignment and Functional Annotation of MAGs

The phylogenetic analysis and taxonomic novelty of MAGs were carried out using Genome Taxonomy Database Toolkit (GTDB-TK) for bacterial and archaeal genomes (Chaumeil et al., 2020) by placing the MAGs into a domain-specific reference tree. The functional metabolic potential of MAGs obtained from both SR1 and BS14 metagenomes was annotated using Distilled and Refined Annotation of Metabolism (DRAM). DRAM annotates the MAGs using different databases, including UniRef90, PFAM, dbCAN, RefSeq, VOGDB, and MEROPS peptide database (Shaffer et al., 2020).

## Results and Discussion

### Taxonomic Profiling and Biodiversity Analysis of BS14 and SR1

**Figure 1:**
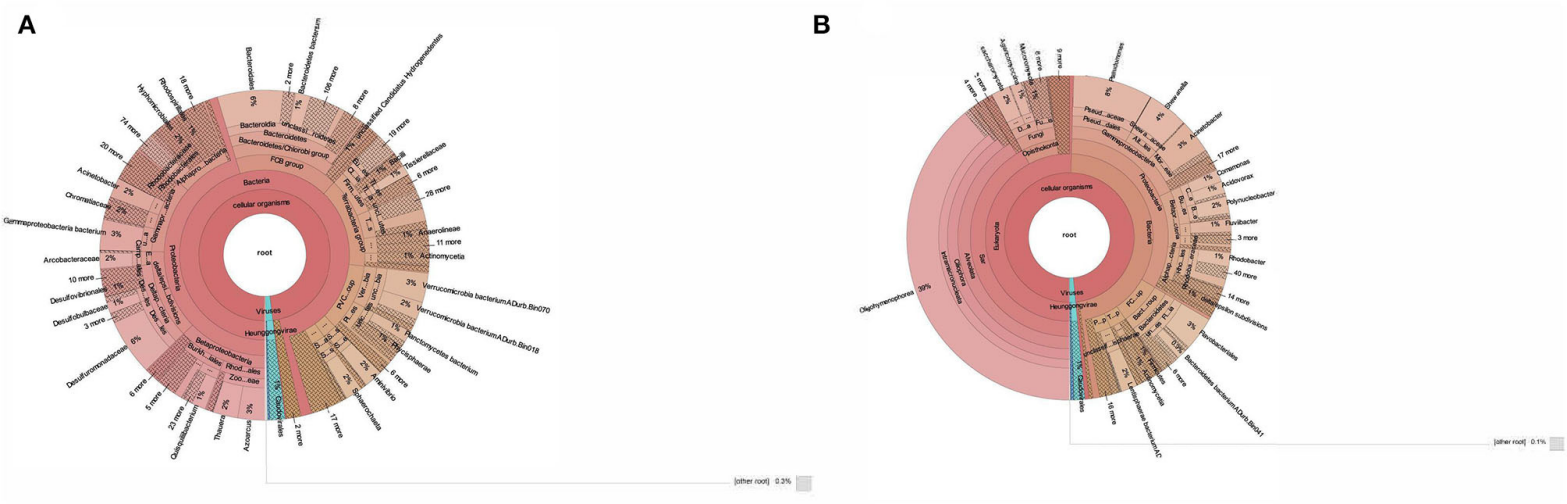
**(A)** Taxonomic classification of BS14 at the genus level in krona plot. **(B)** Taxonomic classification of SR1 at the genus level in krona plot.

The taxonomic profiling of BS14 and SR1 was performed by aligning the contigs using DIAMOND against the nr-database in fast mode and further analyzed using Kaiju. In this study, we observed the dominance of the bacterial domain in the BS14 sample with a prevalence of 96%. We also observed the presence of other domains with an abundance of 0.94%, 0.48%, and 1.7% for archaea, eukarya, and viruses, respectively. In BS14, the predominant phyla observed were Proteobacteria, with an abundance of 47%, and the second predominant phylum was Bacteroidetes (15.3%). The other phyla observed in significant numbers belong to Verrucomicrobia, Firmicutes, Tenericutes, Planctomycetes, Acinetobacter, and others, with an abundance of 6.1%, 6.3%, 3.9%, 3.8%, 1.4%, and 8.4%, respectively. We have also observed the presence of Lentisphaerae, Planctomycetes, Spirochaetes, Synergistetes, Chloroflexi, Thermotogae, and Euryarchaeota (Figure 1A). In the case of BS14, among β-Proteobacteria, Rhodocyclales and Burkholderiales were dominant orders present with a prevalence of 46% and 42.2%, respectively, and other orders, i.e., Neisseriales (5%) and Nitrosomonadales (2.6%), respectively. Desulfuromonadales (59.4%), Desulfobacterales (15.2%), Desulfovibrionales (9.4%), Syntrophobacterales (4.38%), and Myxococcales (1.38%) were abundant order of δ-proteobacteria in BS14 metagenome. Among γ-proteobacteria, most prevalent orders were Moraxellales, Chromatiales, Pseudomonadales, Xanthomonadales, Enterobacteriales, Methylococcales, and Oceanospirillales with an abundance of 18.9%, 18%, 6.16%, 5.1%, 3.3%, 2.4%, and 2.2%, respectively. In the case of α-proteobacteria, Rhodobacterales (29.5%), Hyphomicrobiales (29.3%), Rhodospirillales (12.7%), Sphingomonadales (10%), and Caulobacterales (7%) were observed. Campylobacteriales (98.4%) were the most prevalent order observed in the case of ε-proteobacteria in BS14.

In contrast, SR1 has a high prevalence in the Eukarya domain, with an abundance of 50.4%. The other domains, i.e., bacteria, archaea, and viruses, were observed at 47.2%, 0.36%, and 1.4%, respectively (Figure 1B). Ciliophora (eukarya), with 79.3%, was observed as the predominant phyla in SR1, and other dominant phyla (eukarya) observed in SR1 were Ascomycota, Basidiomycota, Mucormycota, and Chlorophyta ~2.7%, 2.0%, 1.16%, and 0.95%, respectively. Among Ciliophora, the Oligohymenophora (97%) was observed as the most predominant class, and the other classes observed in Ciliophora involved Spirotrichea (1.3%), Heterotrichea (1.2%), Litostomatea (0.18%), and Armphora (0.025%).

In BS14, 0.94% of the total sequences were assigned to archaea, with 65% and 25% of the sequences assigned to Euryarchaeota and Candidatus phyla. Other archaeal phyla in BS14 are Thaumarchaeota, Crenarchaeota, and Nanoarchaeota, with an abundance of 2.1%, 1.59%, and 0.7%, respectively. In comparison, 0.36% of total sequences were assigned to archaea in SR1, among which Euryarchaeota and Candidatus phyla were also found with a prevalence of 38.3% and 24.3%, respectively. The Crenarchaeota was also found in SR1 at ~7.5%.

The eukarya domain of BS14 indicated the presence of Ascomycota and Basidiomycota as dominant phyla with an abundance of 32% and 21.8%, and the other eukaryotic phyla, i.e., Chlorophyta (9.2%) and Mucormycota (7.1%), were also present. Among viruses, 85% of the sequences were assigned to Uroviricota.

In SR1, the taxonomic distribution at the phylum level of the bacterial kingdom indicated the dominance of proteobacteria with an abundance of 69.8% and other phyla, Bacteroidetes, Lentisphaerae, Firmicutes, and Verrucomicrobia, with a prevalence of 14.5%, 3.9%, 2.38%, and 0.94%, respectively (Figure 1B).

The SR-1 was rich in gamma-proteobacteria with an abundance of 53.6%, and the other classes of proteobacteria, i.e., alpha, beta, delta, and epsilon proteobacteria, were observed at 14.6%, 25.4%, 0.198%, and 2.51%, respectively. In contrast, beta-proteobacteria (28%) was found abundant in BS14, and delta (24%), gamma (23%), alpha (17.5), epsilon (3.9%) proteobacteria were observed. Bacteroidias dominated BS14 (51.6%), while SR1 had only 8.9% of the population.

For γ-proteobacteria class of SR1, Pseudomonadales (45%), Alteromonadales (23.6%), and Moraxellales (22.9%) were the predominant orders. For β-proteobacteria, the most prevalent orders obtained were Burkholderiales (59.8%), Rhodocyclales (19.7%), and Neisseriales (14.29). For α-proteobacteria, the Rhodobacterales (76.4%), Rickettsiales (64%), and Hyphomicrobiales (8.5%) were observed as the most abundant orders.

The taxonomic distribution at the species level indicated 7,973 and 3,588 unique assigned species, and 931 and 611 unique classifications were found unassigned in BS14 and SR1, respectively. BS14 was found to be enriched in *Desulfuromonas acetexigens, Synergistetes bacterium, Macellibacteroides fermentans, Arcobacter ellisii, Desulfobulbus propionicus, Desulfovibrio desulfuricans, Azoarcus communis, Mesotoga infera, Parabacteroides chartae, Thauera propionica, Quisquillibacterium transsilvanicum, Bacteroidetes bacterium, Lentimicrobium saccharophilum, Gammaproteobacterium bacterium, Verrucomicrobia bacterium*, and *Aminivibrio pyruvatiphilus*. In SR1, *Pseudomonas fragi, Pseudomonas aeruginosa, Lentisphaerae bacterium, Acinetobacter kyonggiensis, Acinetobacter bohemicus, Fluviibacter phosphoraccumulans, Acidovorax temperans, Paenirhodobacter* sp. MME-103, *Aliarcobacter cryaerophilus, Comamonas aquatica, Shewanella baltica, Arcobacter cryaerophilus, Rhodocyclaceae bacterium, Neisseriaceae bacterium, Polynucleobacter yangtzensis, Rickettsiales bacterium*, and *Rhodobacter* spp. were observed as most abundant species. As SR1, metagenome was predominated by eukarya; the most abundant species were *Tetrahymena thermophila, Ichthyophthirius multifiliis, Pseudocohnilembus persalinus, Stentor coeruleus, Paramecium sonneborni*, and *Stylonychia lemnae*.

The biodiversity analysis of the BS14 and SR1 metagenome was obtained manually. The contigs were assigned with taxonomy *via* Kaiju, and the abundance and proportions were extracted manually for the estimation of the diversity. The BS14 and SR1 samples showed a Simpson's index (Simpson, 1949) of 0.989 and 0.838, respectively. It indicates that both of the sampling sites exhibited a diverse microbial community. Although the SR1 sample was found to be abundant in eukaryotes, given it is a natural river habitat as compared with the sludge environment of BS14. The Shannon-Weiner index (Shannon, 1948) of 6.53 and 4.44 again supports the diverse microbial profile of the BS14 and SR1 sites, and the maximum allowed diversity for both was found to be 8.98 and 8.18, respectively. The evenness of 0.72 and 0.54 suggests that the population of BS14 was evenly distributed and that of SR1 was found to be quite low. Given the diversified nutritional value of the sludge at the BS14 site, the population was getting a constant supply of all basic nutrients to sustain an even and diverse growth, which was found to be lacking in the natural river (SR1).

The alpha diversity of the BS14 and SR1 was manually estimated from the taxonomic units of the metagenome assigned through Kaiju. The species-level diversity analysis revealed an alpha diversity of 7,973 and 3,588 unique species out of 63,471 and 35,952 contigs in BS14 and SR1 samples, respectively. The beta diversity and beta diversity index were calculated to be 8,793 and 0.239, with 1,384 common species among both the samples, respectively. The predicted gamma diversity was depicted as around 10,177 unique species in both samples.

### Functional Analysis of BS14 and SR1

To determine the functional potential of BS14 and SR1, we annotated their predicted gene sequences against the KEGG database using the freely available webserver GhostKOALA. KEGG database is a repository of metabolic pathways to unravel the metabolism, biological pathways, disease, and drugs. For BS14 and SR1, 43.7% and 29.2% of total ORFs were annotated against the KEGG database and assigned to different functional pathways (Supplementary Figure S1). Genetic information processing was the most abundant functional category observed for BS14 and SR1 (15,780 and 7,843 ORFs, respectively) (Supplementary Figure S2).

**Figure 2:**
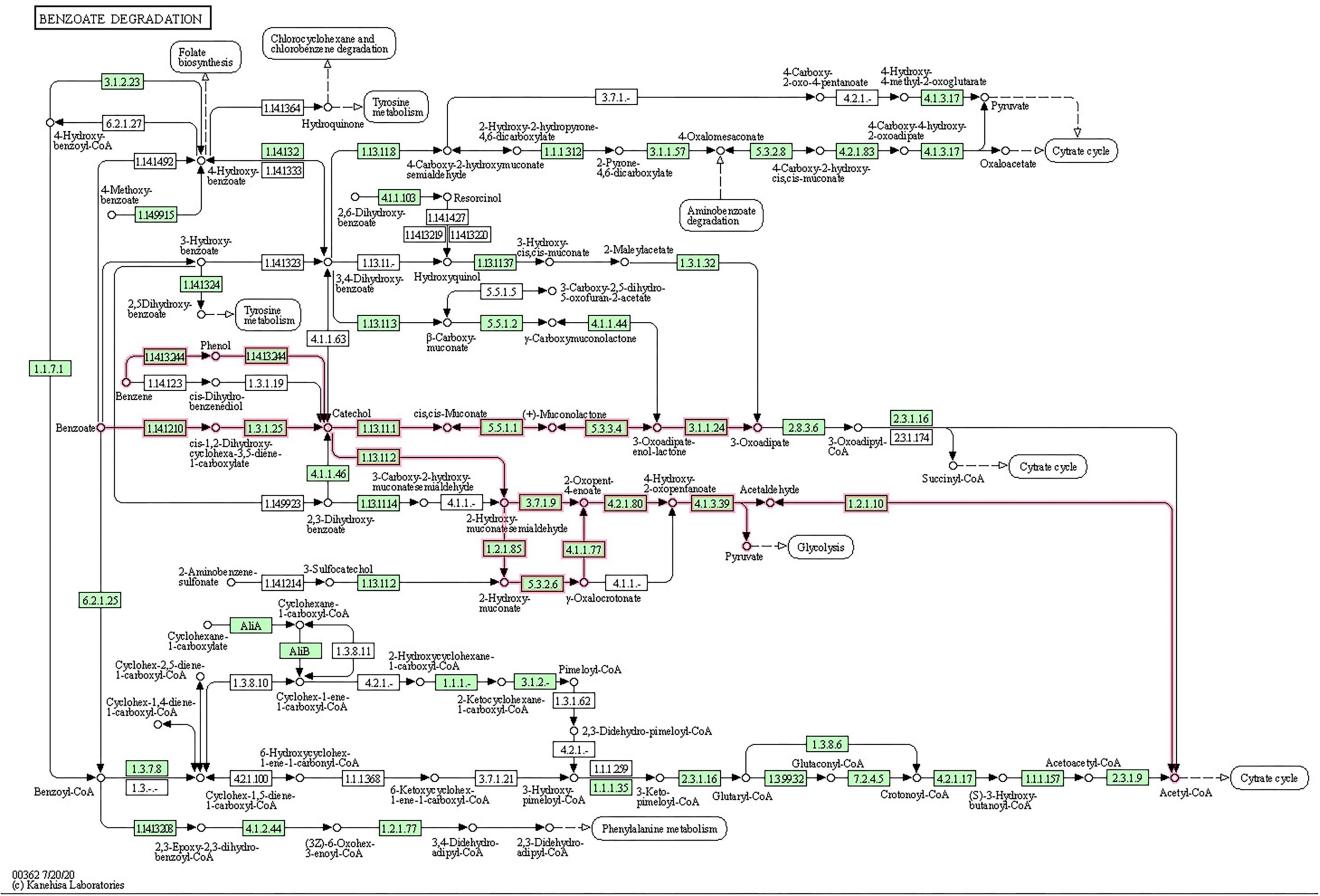
Open reading frames (ORFs) of BS14 and SR1 mapped to benzoate biodegradation pathway.

The metabolic potential of BS14 and SR1 was also analyzed for biodegradation of xenobiotic compounds (Supplementary Figure S3). Most ORFs for BS14 (831) and SR1 (330) were assigned to different KO IDs (70 and 55 for BS14 and SR1, respectively) belonging to benzoate degradation, which also indicated the presence of benzoate as the most prevalent aromatic pollutant of CETP. The complete metabolic pathway was also observed for benzoate degradation in the BS14 and SR1 metagenome (Figure 2). The benzoate undergoes complete degradation with the help of ring hydroxylating and ring cleavage enzymes (Valderrama et al., 2012). The key enzymes responsible for benzoate degradation, i.e., benzoate 1,2 dioxygenase subunit alpha and beta (EC: 1.14.12.10), benzoate 1,2 dioxygenase reductase (EC: 1.18.1), dihydroxycyclohexadiene carboxylate dehydrogenase (EC: 1.3.1.25), and catechol 1,2 dioxygenase (EC: 1.13.11.1) were also observed in both metagenomes.

The functional annotation through KEGG also revealed the abundance of rTCA and chemolithotropism, which involves the energy generation using inorganic compounds, especially sulfur or nitrogen, as electron donors where the level of molecular O_2_ is deficient (Reddy et al., 2019). During analysis, the equal distribution of sulfur and nitrogen metabolism was observed in both BS14 and SR1. The complete sulfur metabolism was observed, including both assimilatory and dissimilatory sulfate reduction, thiosulfate oxidation by SOX complex and amino acid metabolism. The critical enzymes for sulfur metabolism, i.e., dissimilatory sulfite reductase (EC: 1.8.99.5), adenylylsulfate reductase, subunit A (EC:1.8.99.2), 3′-phosphoadenosine 5′-phosphosulfate synthase (EC: 2.7.7.4), phosphoadenosine phosphosulfate reductase (EC: 1.8.4.8), and sulfate reductase (EC: 1.8.1.2;1.8.1.7) were found in sulfur metabolism pathway. Similarly, the complete nitrogen metabolism, i.e., nitrate reduction and denitrification, were observed in both metagenomes with the presence of key enzymes, i.e., nitric oxide reductase subunit B (EC: 1.7.2.5), nitrous oxide reductase (EC: 1.7.2.4), nitrogenous molybdenum iron protein alpha chain (EC: 1.18.6.1), nitrate reductase (EC: 1.7.5.1), and nitrite reductase (EC: 1.7.2.1).

The functional analysis of both metagenomes also revealed the presence of necessary CO_2_ assimilatory enzymes involved in rTCA cycle, i.e., aconitate hydratase (EC: 4.1.2.3), isocitrate dehydrogenase (EC: 1.1.1.42), 2-oxoglutarate/2-oxo acid ferredoxin oxidoreductase subunit alpha (EC: 1.2.7.3), fumarate hydratase (EC: 4.2.1.2), fumarate reductase (EC: 1.3.5.4; 1.3.5.1), pyruvate carboxylase (EC: 6.4.1.1), malate dehydrogenase (EC: 1.1.1.37), succinyl CoA synthetase (EC: 6.2.1.5), pyruvate-ferredoxin oxidoreductase (EC: 1.2.7.1), and most important enzyme ATP citrate lyase (EC: 2.3.3.8).

Various studies have shown sewage and effluent treatment plants as sources of AMR genes. Hendriksen et al. have shown the impact of municipal sewage treatment and effluent plants on disseminating AMR genes in its nearby freshwater system (Hendriksen et al., 2019). The KEGG analysis of BS14 and SR1 by GhostKOALA also indicated the prevalence of unique KO terms representing antimicrobial resistance. The resistance against the beta-lactams, cationic antimicrobial peptides (CAMP), and vancomycin was rich in BS14 and SR1. During the KEGG analysis, we observed that 996 ORFs of BS14 metagenome were mapped against 31 unique KO IDs belonging to beta-lactam resistance profile, 786 ORFs against 23 KEGG orthology terms related to CAMP resistance genes, and 457 ORFs related to 9 unique KO IDs of vancomycin resistance genes. Similarly, for SR1, the most prevalent resistance modules were observed against beta-lactam antibiotics, CAMP, and vancomycin. For SR1, 358, 336, and 117 ORFs were assigned to beta-lactam, CAMP, and vancomycin, respectively.

The complete metabolic module for beta-lactam resistance is shown in (Supplementary Figure S4). Various resistance modes were observed in BS14 and SR1 for beta-lactam resistance. The resistance modes include the loss or severe reduction of porins of outer membrane encoded by ompU, ampG; coding for MES transporter, nagZ; beta-N-acetylhexosaminidase (EC: 3.2.1.52), ampR; regulator of gene expression of beta-lactamase, ampC; beta-lactamase class C, oppA; oligopeptide transport system substrate-binding proteins, blaI; penicillinase repressor, bla2; beta-lactamase classA, penicillin-binding proteins mrcA, mrcB, pbp2A, mrdA, and fts1. The multidrug efflux pumps, i.e., arcA, mexA, ade1, smeD, mtrC, cmeA, adeA, and adeB for beta-lactam antibiotics, were also observed in BS14 and SR1. The beta-lactamase class A, class B, class C, and class D (EC; 3.5.2.6) were also observed.

**Figure 3:**
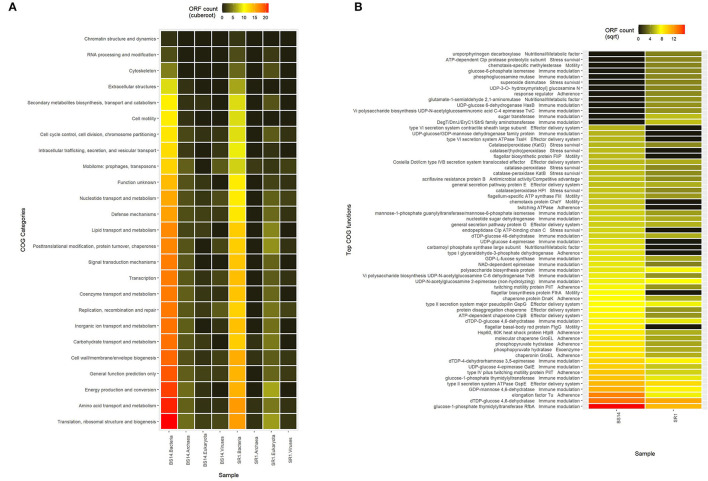
**(A)** Heatmap showing the Cluster of Orthologous Genes (COG) functional assignment of BS14 and SR1 at the Kingdom level and **(B)** heatmap showing the top 50 COG functional assignments of BS14 and SR1.

**Figure 4:**
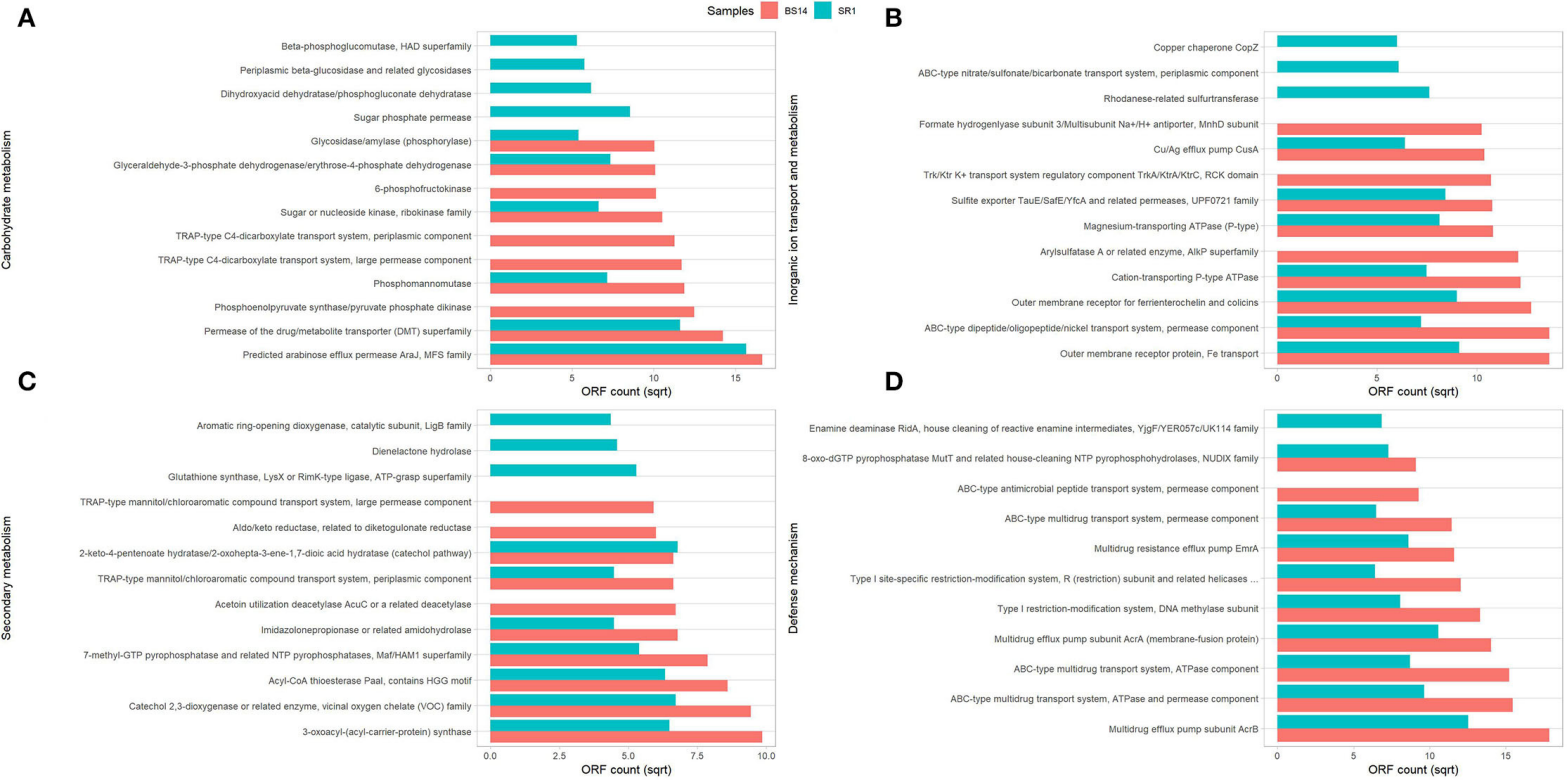
Representation of top 10 abundant features of Clusters of Orthologous Genes (COG) category for BS14 and SR1 involved in **(A)** carbohydrate metabolism; **(B)** inorganic ion transport and metabolism; **(C)** secondary metabolism; and **(D)** defense mechanism.

The predicted ORFs of BS14 and SR1 samples were aligned using DIAMOND against the COG database, and a total of 95,907 and 42,017 functions were identified from the BS14 and SR1 metagenome, respectively. The COG functions were categorized as shown in (Figure 3A), which made it easy to understand that the bacterial community majorly contributed to the functional potential of the system, followed by eukaryotes in SR1. The top 50 COG functions of BS14 and SR1 (Figure 3B) indicate the abundance of properties such as lipid metabolism, defense mechanism, transcription and translation regulation, cell wall biogenesis, signal transduction, etc. Certain enzymes such as transposase (0.56%), DNA binding response regulator or osmoregulatory protein (OmpR family; 0.44%), glycosyltransferase (0.49%) for cell wall synthesis, short-chain alcohol dehydrogenase (0.49%), and multidrug efflux pump (AcrB; 0.33%) were dominant in BS14 metagenome. The features of SR1 (Figure 3B) were similar to the BS14 metagenome. Although the predicted taxonomical dominance of SR1 was covered by the eukaryotes (especially Tetrahymena), it exhibited equal or higher relative abundance of COG functions with respect to BS14. SR1 was also prevalent in DNA binding transcription regulator (LysR family; 0.94%), arabinose efflux permease (0.58%), short-chain alcohol dehydrogenase (0.53%), DNA binding response regulator or osmoregulatory protein (OmpR family; 0.54%), multidrug efflux pump (AcrB; 0.37%), etc. The COG functions of BS14 and SR1 were additionally categorized into carbohydrate metabolism (6.37, 5.55%), inorganic ion transport and metabolism (6.22, 6.53%), secondary metabolism (1.36, 1.57%), and defense mechanism (4.06, 3.69%), and the top 10 enzymes are shown in (Figure 4). In BS14 and SR1, the carbohydrate metabolic category exhibited a high count of arabinose efflux permease (0.28, 0.58%), permease of drug/metabolite transporter (DMT) superfamily (0.21, 0.32%), phosphoenolpyruvate synthase (0.16, 0%: zero indicates low abundance), and enzymes for other carbohydrates (Figure 4A). The inorganic ion transport and metabolism showed the presence of outer membrane receptor for Fe-transport (0.19, 0.19%) and for ferrienterochelin and colicin (0.16, 0.19%), cation transporting P-type ATPase (0.15, 0.13%), and other metal/inorganic ion transporters (such as copper, potassium, nickel, sulfur, magnesium, sulfite, and nitrate/nitrite) (Figure 4B). The secondary metabolic category showed the prevalence of catechol 2,3-dioxygenase (0.09, 0.1%), 3-oxoacyl synthase (0.1, 0.09%), acyl-CoA thioesterase (0.077, 0.095%), and other enzymes (Figure 4C) of central aromatic metabolic pathways for benzoate and aminobenzoate utilization as shown in (Figure 2). The category of defense mechanism was abundant in multidrug efflux pump (AcrB; 0.33, 0.37%), ABC-type multidrug transport system-ATPase component (0.24, 0.18%), permease component (0.24, 0.22%), and other efflux system for peptides, etc. (Figure 4D).

The ORFs of BS14 and SR1 were also aligned against the PFAM database (Mistry et al., 2021) with the help of the SqueezeMeta pipeline, and the total complete functional profile was extracted with the inbuilt SQM tool of the software. An average of 7,292 and 6,870 unique functions were assigned to the BS14 and SR1 samples. In the BS14 sample, 23% and 25% of the ORFs were unmapped and unclassified respectively, whereas in SR1, 24% and 21% of the ORFs were unmapped and unclassified respectively. The samples were prevalent in the proteins responsible for the ATP binding, phosphorelay signal transduction system, membrane integrity, transmembrane transport, biosynthetic process, catalytic activity, oxidoreductase activity, carbohydrate metabolic process, etc. The top 50 functional assignment is shown in a heatmap in (Supplementary Figure S5).

### Antimicrobial Resistance Profiling of BS14 and SR1

The alignment of BS14 and SR1 ORFs against the CARD database (Alcock et al., 2020) yielded 33 and 32 unique resistance profiles against different types of antibiotics. Some of them exhibited multidrug resistance properties. In BS14, the majority (11%) of ORFs showed resistance to the cephalosporin class of antibiotics. Second major (7.2%) resistance was found against the penam and several other classes of beta-lactam antibiotics such as carbapenem (5.6%), penem (2.84%), and monobactam (3.51%). In total, 3,872 copies of AMR genes were found in the BS14 metagenome, where most of them targeted beta-lactams and few against fluoroquinolones (8.96%), macrolides (8.13%), tetracycline (8.08%), phenicols (7.6%), aminoglycosides (6.19%), etc. In SR1, the beta-lactam class of cephalosporin and penam constitutes 13% and 11% of the AMR content, respectively, followed by tetracycline (8.7%), fluoroquinolone (7.7%), carbapenem (7.6%), macrolide (7.2%), etc. In SR1, 2,447 copies of AMRs were found and shared a similar profile with BS14. The shared resistome profile of both BS14 and SR1 indicated the dissemination of AMR genes from CETP to its receiving water body.

Out of 35 unique resistomes, SR1 was found to lack the gene for mupirocin, glycopeptide, and bicyclomycin, whereas BS14 lacked the benzalkonium chloride and fosfomycin.

**Figure 5:**
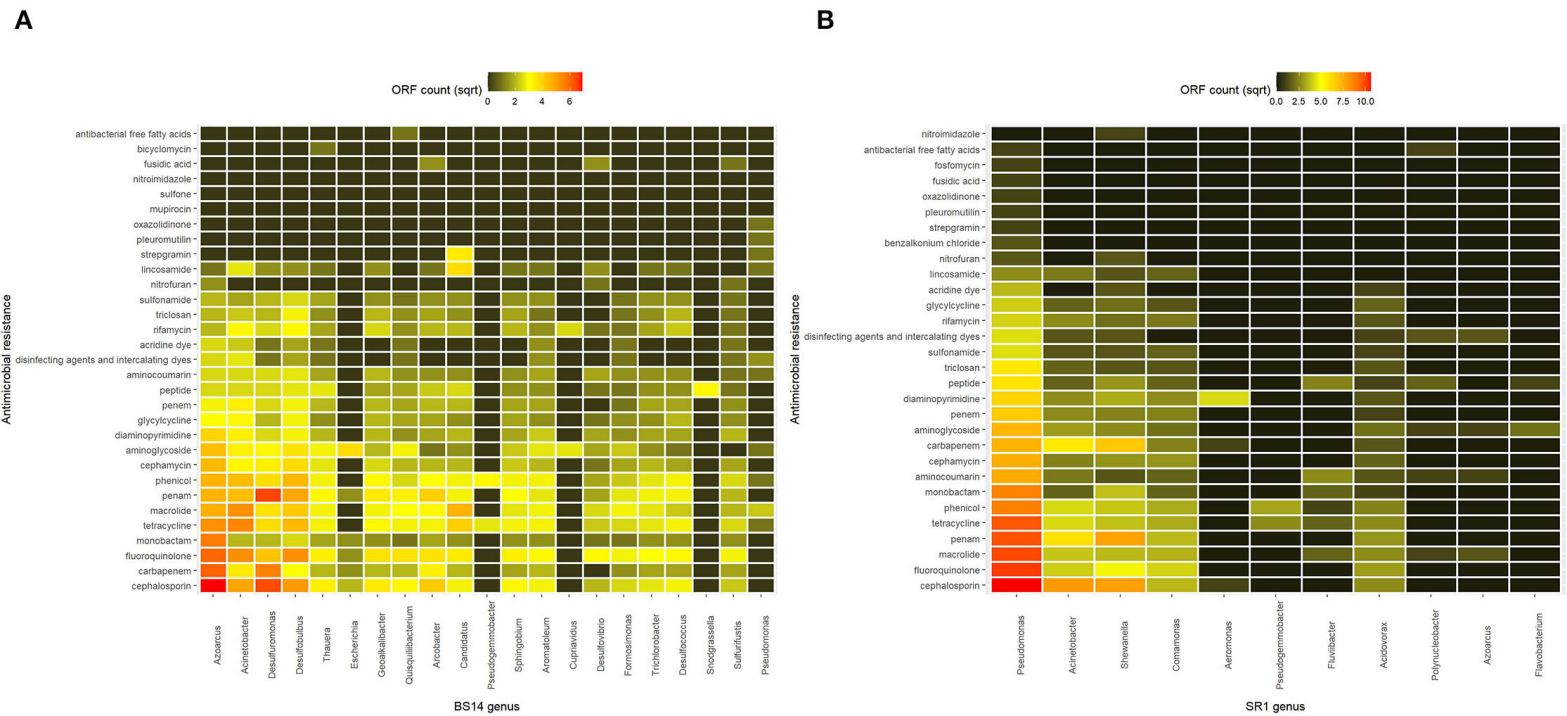
Heatmap showing the antimicrobial resistance (AMR) prevalence of **(A)** BS14 sample at the genus level and **(B)** SR1 sample at the genus level.

The taxonomic annotation of the CARD data was performed manually, and the classification was assigned from Kaiju to analyze AMRs at the genus level. It was found that in the BS14 sample, the majority of AMRs were prevalent in *Azoarcus* (9.2%), *Acinetobacter* (6.9%), *Desulfuromonas* (6.3%), *Desulfobulbus* (6.4%), *Candidatus* (3.5%), and *Arcobacter* (3.1%). The rest of the AMRs in BS14 was originating from *Thauera* (2.8%), *Geoalkalibacter* (2.9%), *Quisquiliibacterium* (2.4%), *Sphingobium* (2.3%), *Aromatoleum* (2.3%), *Desulfococcus* (2.1%), *Trichlorobacter* (1.9%), *Formosimonas* (1.6%), *Sulfurifustis* (1.5%), *Desulfovibrio* (1.3%), *Escherichia* (0.67%), *Pseudogemmobacter* (0.4%), *Cupriavidus* (0.33%), *Snodgrassella* (0.26%), and *Pseudomonas* (0.39%). Approximately 40.05% of the AMR was not assigned to a genus (Figure 5A). Comparatively in SR1, *Pseudomonas* harbored ~45.8% of the total AMR content, and the rest was distributed among *Acinetobacter* (10.6%), *Shewanella* (12.6%), *Comamonas* (5.3%), *Aeromonas* (0.81%), *Pseudogemmobacter* (0.69%), *Fluviibacter* (0.93%), *Acidovorax* (2.8%), *Polynucleobacter* (0.36%), *Azoarcus* (0.3%), and *Flavobacterium* (0.25%) (Figure 5B).

### Virulence Profiling of BS14 and SR1

**Figure 6:**
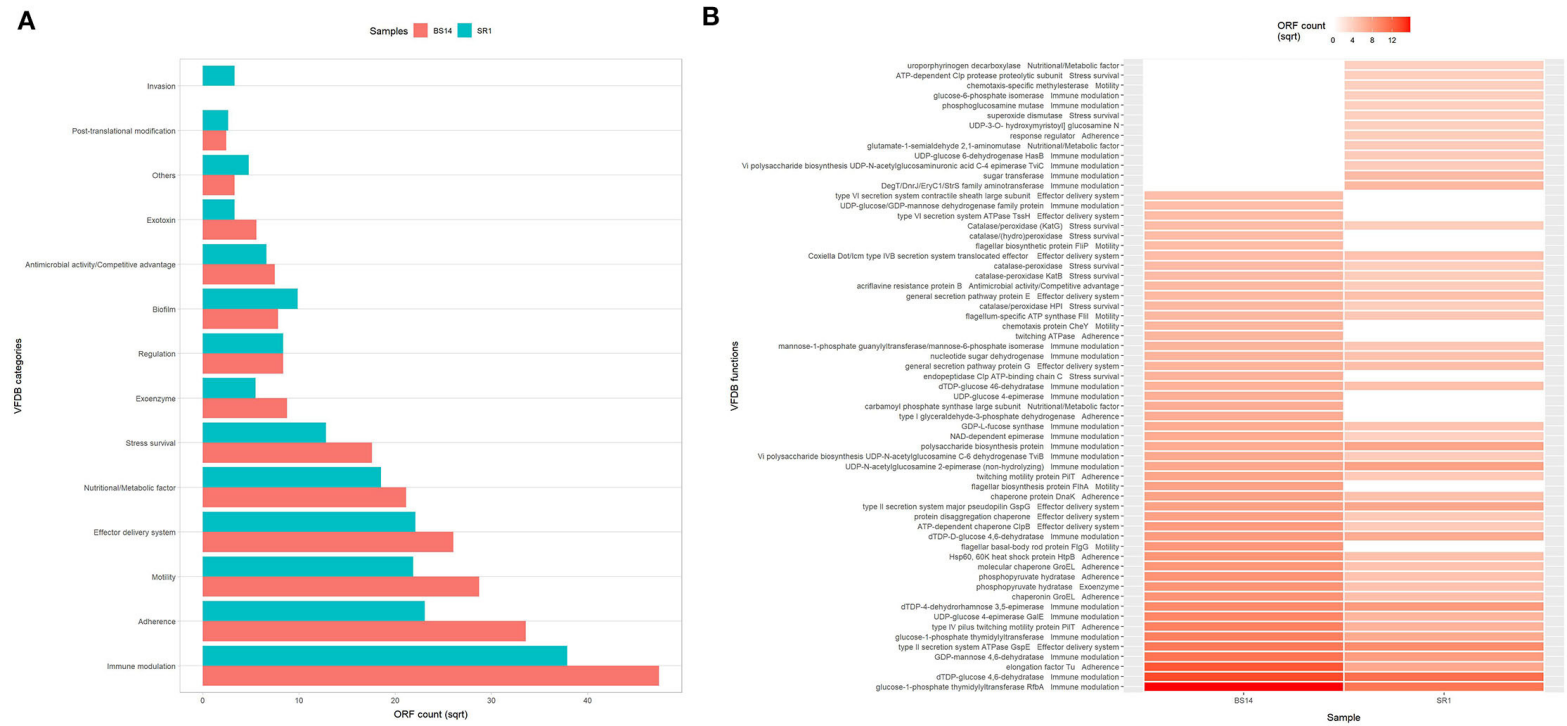
**(A)** Distribution of virulent categories among the BS14 and SR1 samples from virulence factor database (VFDB) alignment and **(B)** heatmap showing the top 50 abundant virulent factors of BS14 and SR1 sample.

**Figure 7:**
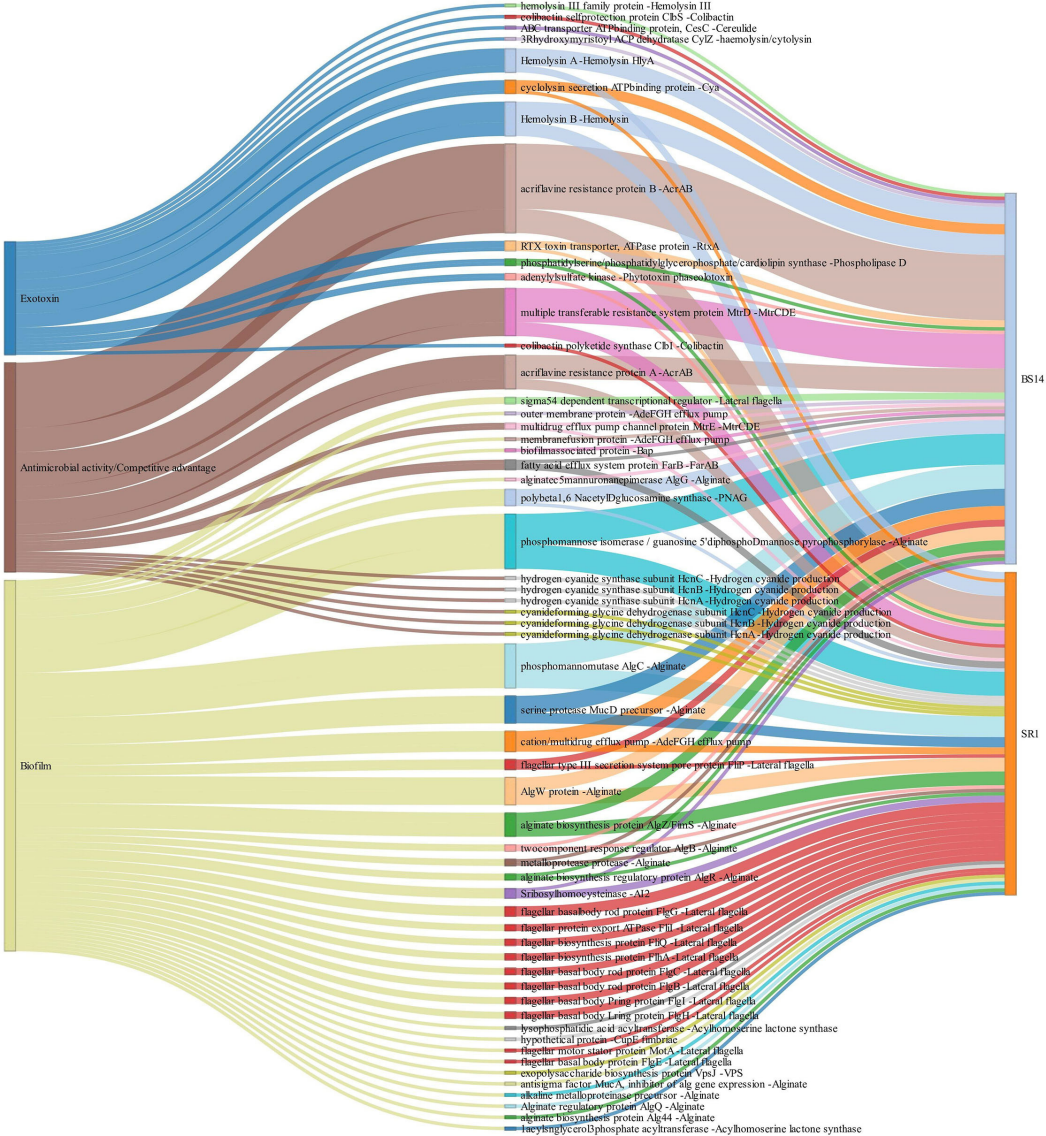
Exploration of the biofilm, antimicrobial activity/competitive advantage, and exotoxin category of VFDB in BS14 and SR1 samples.

The alignment of BS14 and SR1 ORFs with the virulence factor database (VFDB) yielded 5,953 and 3,736 functions after removing the redundancy. Each ORFs of an individual node were assigned with the taxonomic annotation to determine the dominant class and genus contributing to the extent and type of virulence. In the BS14 class level, the majority of virulence was found to be distributed among the Betaproteobacteria (21%), Gammaproteobacteria (19.62%), Deltaproteobacteria (18.52%), Alphaproteobacteria (11.01%), Bacteroidia (3.61%), Clostridia (1.42%), etc. At the genus level, the abundance of *Desulfuromonas* (6.81%), *Thauera* (6.38%), *Azoarcus* (5.35%), *Acinetobacter* (4.48%), *Geoalkalibacter* (3.25%), and *Desulfobulbus* (1.86%) was observed, whereas the major fraction was not assigned (38.65%) with a taxonomy. At the SR1 class level, the majority belonged to Gammaproteobacteria (51.04%), Betaproteobacteria (27.7%), Flavobacteriia (5.72), Alphaproteobacteria (5.46%), Oligohymenophorea (0.91%), etc. It is observable in SR1 that despite having a 50% population of eukaryotes, the majority of virulence is sourced from the prokaryotes. On exploring at the genus level, *Pseudomonas* (30.91%) contributed to more virulent factors followed by *Shewanella* (9.71%), *Acinetobacter* (9.28%), *Polynucleobacter* (7.36), *Flavobacteriia* (3.88%), *Fluviibacter* (3.37%), etc. The virulent factors of BS14 and SR1 were further classified into categories (Figure 6A) as immune modulation (37.79, 38.46%), adherence (18.96, 14.23%), motility (13.87, 12.79%), effector delivery system (11.4, 13.08%), nutritional metabolic factor (7.5, 9.18%), stress survival (5.2, 4.38%), exoenzyme (1.29, 0.8%), regulation (1.17, 1.87%), biofilm (1.02, 2.59%), posttranslational modification (0.1, 0.18%), invasion (0, 0.29%), exotoxin (0.52, 0.29%), antimicrobial activity/competitive advantage (0.94, 1.17%), and others (0.18, 0.61%), and some top 50 functions of VFDB as shown in (Figure 6B) indicates the presence of factors promoting the cell membrane modulation, adherence, motility, secretion system, antimicrobial resistance, radical scavenging, etc. The mining of exotoxin revealed the presence the of hemolysin A and B in both the samples, hemolysin III in BS14, and colibactin (clbI; a genotoxic metabolite) (Balskus, 2015) in SR1 but BS14 had colibactin self-protection protein (clbS) (Tripathi et al., 2017) and a gene for cyclolysin secretion protein (Cya) in both samples as shown in (Figure 7). The antibacterial activity/competitive advantage category contained the genes for hydrogen cyanide production (HcnABC; in SR1 only), resistance against acriflavine (AcrAB; in BS14 and SR1), and efflux pumps as shown in (Figure 7). The category of biofilm was also explored to reveal the genes responsible for localization and movement (flagellar), adherence, and mucus secretion (Figure 7).

### Carbohydrate Active Enzyme Profiling of BS14 and SR1

**Figure 8:**
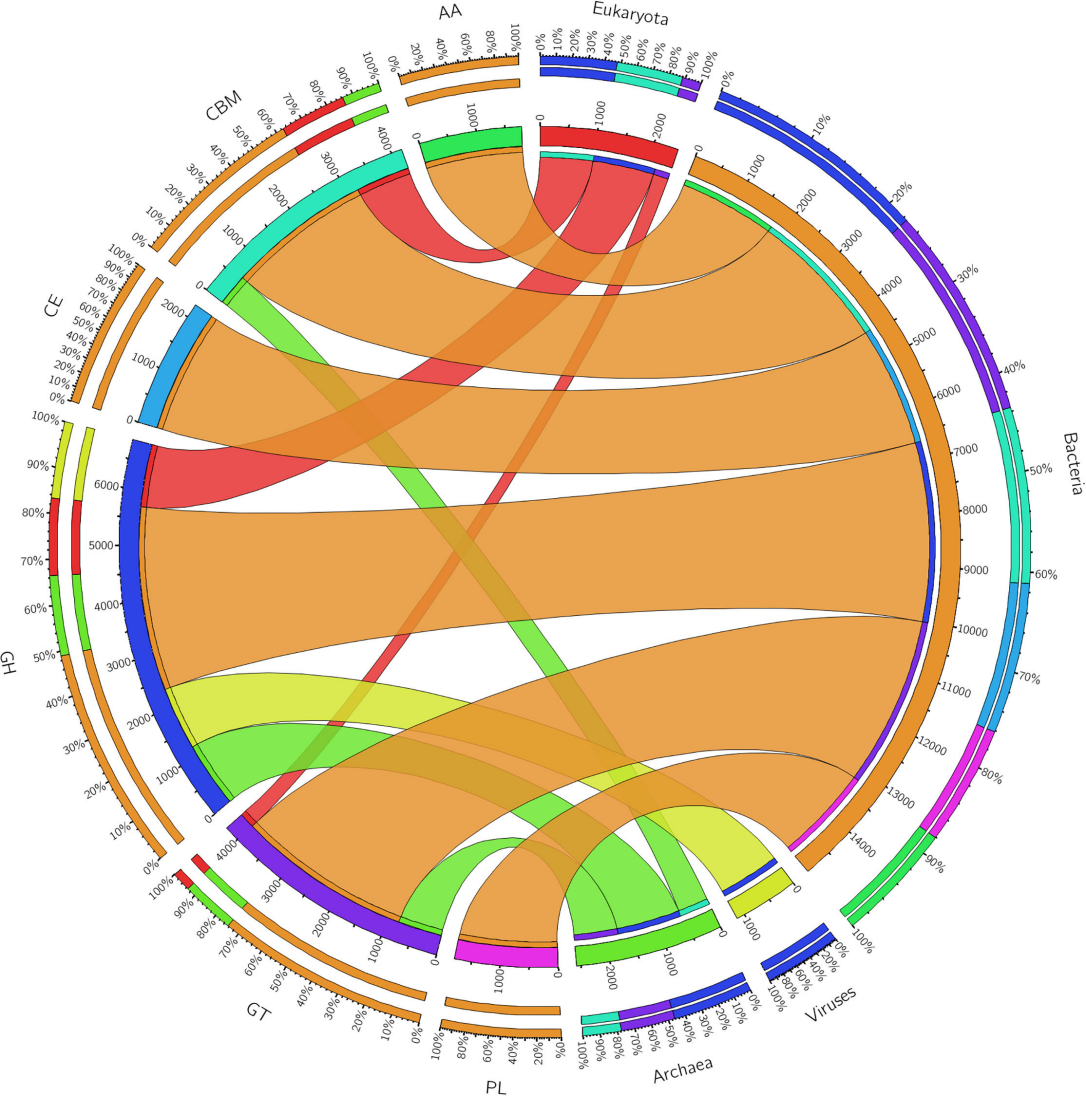
Circos plot representation of the carbohydrate-active enzymes of BS14 at the Kingdom level.

**Figure 9:**
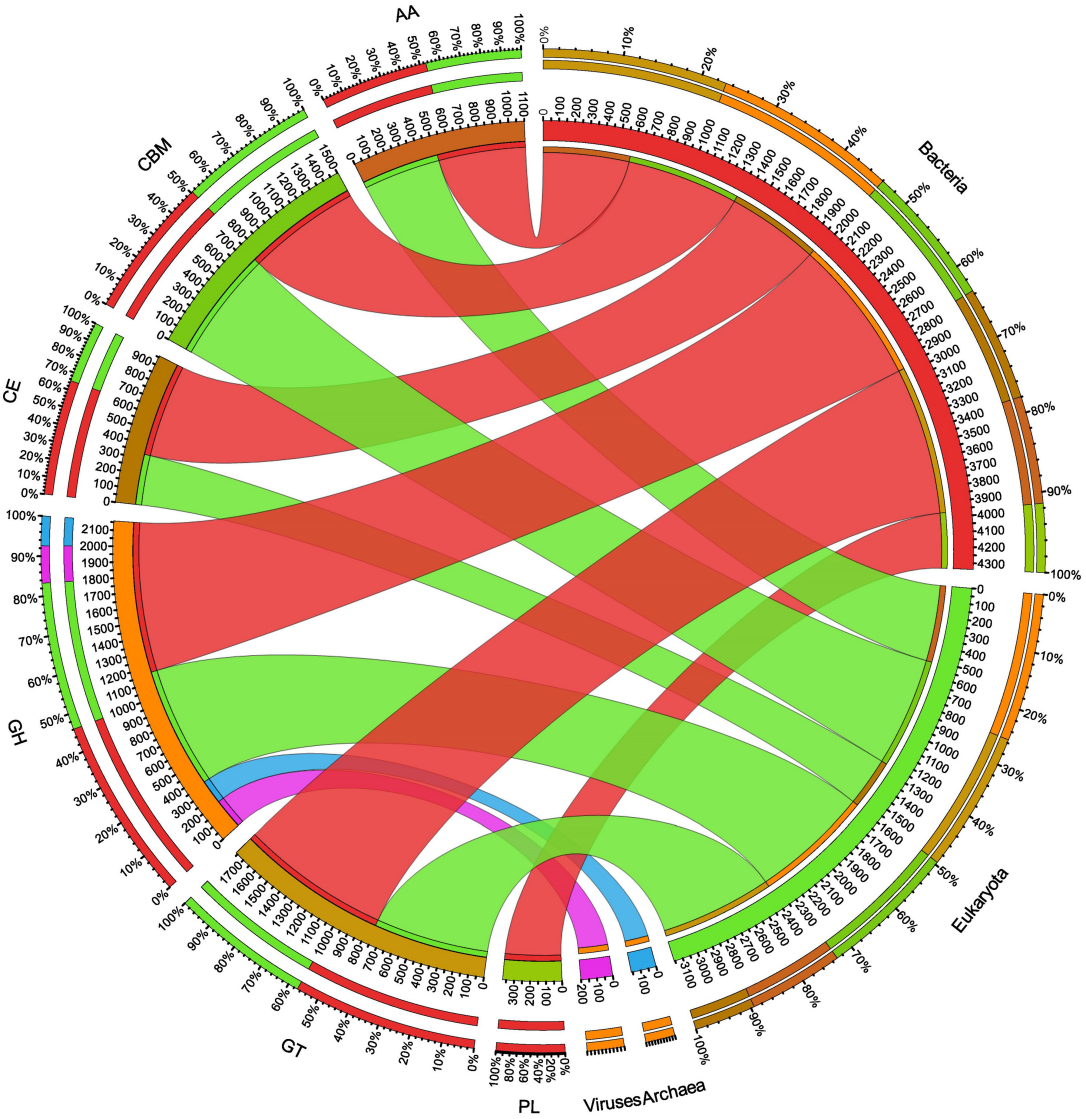
Circos plot representation of the carbohydrate-active enzymes of SR1 at the Kingdom level.

The ORFs of BS14 and SR1 metagenomes were aligned against the CAZy database (Drula et al., 2022) with a minimum identity of 50% and query coverage of 90%. A total hit of 6,611 and 2,941 active enzymes was identified in BS14 and SR1 samples with a minimum bit score >50. The classes of enzymes in the CAZy database are distributed as glycosyl hydrolases (GH), glycosyltransferases (GT), polysaccharide lyases (PL), carbohydrate esterases (CE), auxiliary activities (AAs), and carbohydrate-binding module (CBM). In the BS14 sample, most enzymes belonged to the GH and GT classes having an abundance of 51.5% and 33.6%, respectively. The other enzymes, i.e., PL, CE, AA, and CBM, had an abundance of 1.1%, 3.5%, 1.1%, and 8.9%, respectively. The SR1 sample was abundant, with GH and GT covering 40.9% and 40.5% of the total active enzymes. The other classes, i.e., PL, CE, AA, and CBM occupied 0.4%, 2.8%, 3%, 12.1%, respectively. The taxonomy assignment by Kaiju showed that around 98.65% and 77.52% of active enzymes in BS14 and SR1 belonged to the bacterial kingdom, respectively. Since the SR1 sample had a higher abundance of eukaryotes, it represented 22.2% of the active enzymes. The circos plot of BS14 and SR1 shows the distribution of carbohydrate-active enzymes of BS14 and SR1 samples at kingdom level classification (Figures 8, 9). The phyla of Proteobacteria and Bacteroidetes held 37.2% and 48.3% and 23.6% and 18.8% of the active enzymes in the BS14 and SR1 samples, respectively.

The top 50 specific carbohydrate-active enzymes were also extracted from the SqueezeMeta tool, where the BS14 and SR1 ORFs were assigned the functional annotation from the KEGG database. BS14 and SR1 showed a common prevalence of lipid, formate, transketolase, glycogen, glucose, citric acid cycle intermediates, and polyhydroxyalkanoate (PHA) metabolism with different relative abundances. SR1 was also metabolically active for chitin. (Supplementary Figures S6A,B) shows the distribution of carbohydrate-active enzymes in the BS14 and SR1 samples.

**Figure 10:**
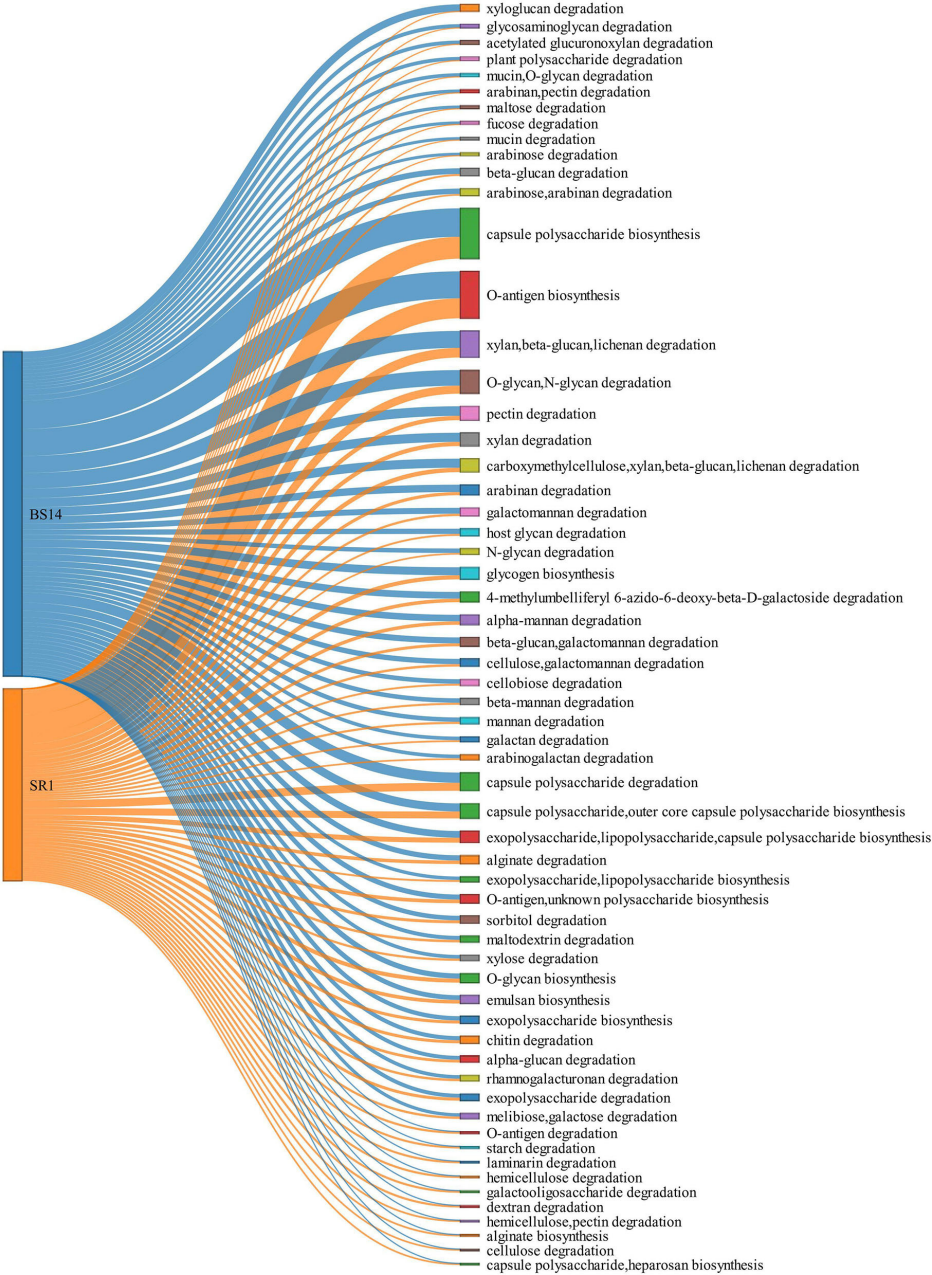
Sankey plot elaborating the total PULDB functions of BS14 and SR1.

**Figure 11:**
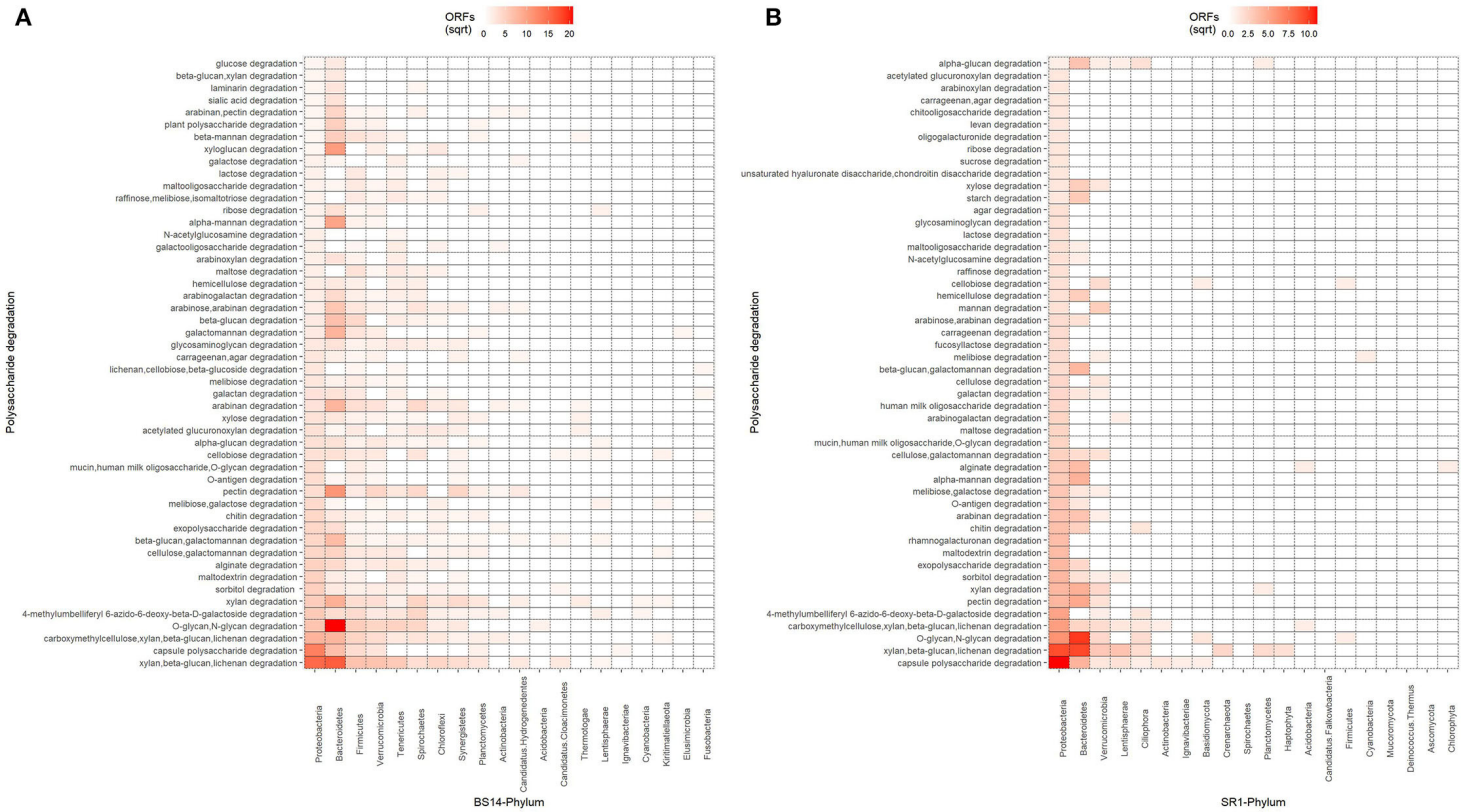
Heatmap showing the distribution of polysaccharide metabolism of **(A)** BS14 and **(B)** SR1 at the phylum level.

The ORFs extracted from BS14 and SR1 were also aligned against the dbCAN-PUL database, and it yielded a total of 8,838 and 3,700 assignments after removing the redundancy. The total PULdb functions (as shown in a Sankey plot) reveal the distribution of polysaccharide biosynthesis and degradation potential among the BS14 and SR1 metagenomes (Figure 10). BS14 and SR1 were abundant in capsule polysaccharide biosynthesis (21.82, 30.78%), O-antigen biosynthesis (19.66, 25.54%), xylan, beta-glucan, lichenan degradation (7.79, 5.86%), O-glycan, N-glycan degradation (6.53, 4.08%), capsule polysaccharide degradation (2.8, 4.08%), and metabolism of some other polysaccharides. The ORFs with PULdb assignments were filtered to extract the polysaccharide degradative potential of BS14 and SR1. These ORFs were also assigned with the taxonomic profile at the phylum level manually using awk. Proteobacteria and Bacteroidetes represented the majority of the functions in both the BS14 and SR1 metagenome but the former was more prevalent in SR1. In BS14, the proteobacteria were highly active in xylan, beta-glucan, lichenan degradation (2.63%), and capsule polysaccharide degradation (1.98%), whereas Bacteroidetes was active in O-glycan, N-glycan degradation (4.88%), xylan, beta-glucan, lichenan degradation (3%), and some other polysaccharides as shown in (Figure 11A). In SR1, the Proteobacterium was a major player in polysaccharide utilization, it was rich in capsule polysaccharide degradation (3.29%), xylan, beta-glucan, and lichenan degradation (2.37%) enzymes, and Bacteroidetes was rich in xylan, beta-glucan, and lichenan degradation (2.45%), and O-glycan and N-glycan degradation (2.72%) (Figure 11B). Several features were found to be in common between the BS14 and SR1 samples, which could indicate the carryover of the functional properties from sludge (BS14) to discharge water (SR1).

### Taxonomic Identification and Functional Annotation of MAGs

During binning, sixteen and thirty MAGs for SR1 and BS14, respectively, were obtained. For downstream analysis, using checkm, the good quality MAGs with >90% completeness and <5% contamination were selected. A total of ten and fifteen good-quality MAGs for SR1 and BS14 were obtained. The checkm profile of MAGs for SR1 and BS14 is given in (Supplementary Tables S1, S2) respectively.

The taxonomy of MAGs for bacterial and archaeal genomes using GTDB-Tk workflow was assigned (Supplementary Tables S3, S4). The results obtained by GTDB classification demonstrated that the MAGs of BS14 were distributed in five different phyla. The eight MAGs of BS14 belong to phylum Proteobacteria, two MAGs were assigned to phylum Bacteroidota, and three MAGs were assigned to Desulfobacterota. Additionally, the phylum Verrucomicrobiota and Firmicutes was also observed. The taxonomic novelty of the MAGs is explained by ANI (average nucleotide identity) value to the closest placed reference as computed by GTDB-Tk workflow (Supplementary Table S5). The MAG, BS14_bin1, and BS14_bin7 were thought to be novel taxa belonging to the family *Syntrophobacteraceae* and *Sphingomonadaceae* with ANI values of 78.49 and 78.5, respectively, with the closest reference genome. Similarly, BS14_bin25 and BS14_bin21 were also formally proposed as novel species belonging to the family *Steroidobacteraceae* and *Geoalkalibacteraceae* with ANI values of 88.91 and 79.08, respectively.

Similarly, in SR1, the major recovered phylum was Proteobacteria, whereas two MAGs were assigned to phylum Bacteroidota, and one MAG was assigned to Verrucomicrobiota and Campylobacter (Supplementary Table S6). In SR1, the ANI value of all the MAGs was higher than the species delineation demarcation cutoff, thus no novel taxa were proposed.

The MAGs were annotated for a functional assignment using DRAM. DRAM is used to profile the MAGs for different metabolism, e.g., carbon, nitrogen, sulfur, and methanotrophy. We compared the MAGs obtained from SR1 and BS14 to analyze their metabolic potential in the microbiome (Supplementary Figures S7A,B).

**Figure 12:**
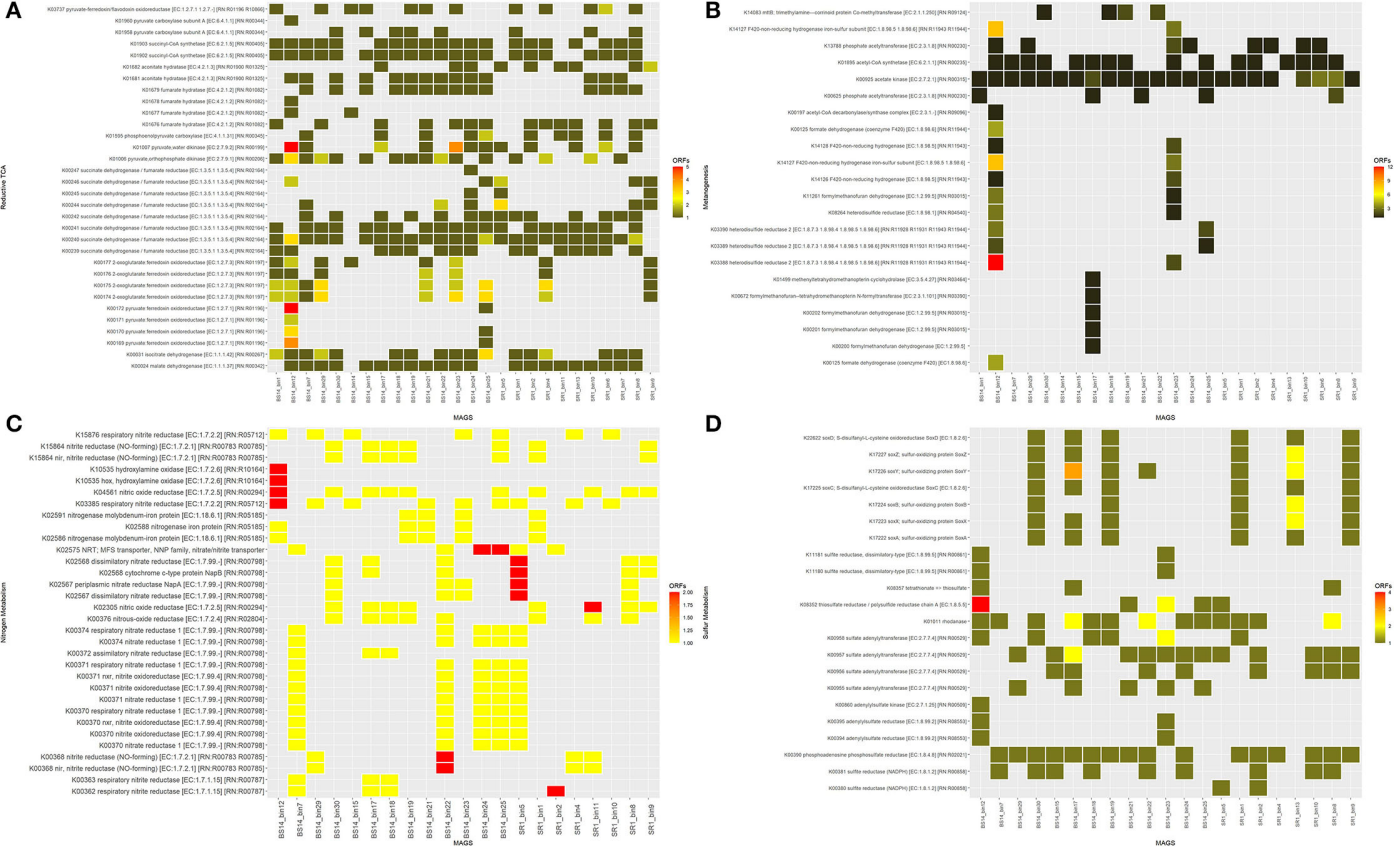
Functional annotation of metagenome-assembled genomes (MAGs) (BS14 and SR1) for the presence of crucial enzymes involved in **(A)** reductive TCA; **(B)** nitrogen metabolism; **(C)** sulfur metabolism; and **(D)** methanogenesis.

The functional annotation using DRAM indicated the presence of various complete energy metabolism pathways in both SR1 and BS14. Most of the MAGs obtained from the SR1 and BS14 showed complete energy metabolism for glycolysis, pentose phosphate pathway, citrate cycle, reductive pentose phosphate pathway, and reductive citrate cycle, whereas the MAGs obtained from BS14 had a high abundance of the Entner-Doudoroff pathway than SR1. All MAGs were compared for the presence of enzymes participating in rTCA pathway and observed their predominance in MAGs obtained from BS14 (Figure 12A). Among the bins of BS14, BS14_bin12 belonging to Desulfobacterota had a high abundance of rTCA enzymes.

The nitrogen and sulfur metabolism presence was also observed in the MAGs of SR1 and BS14, which indicated the diverse biogeochemical cycles prevailing in the niche. Nitrogen and sulfur metabolism plays a crucial role in chemolithotropism by acting as electron donors and mostly prevailing in harsh conditions (Osburn et al., 2014). The enzymes responsible for nitrogen metabolism were equally distributed among the MAGs of BS14 and SR1 (Figure 12B), whereas for sulfur metabolism, the BS14_bin12 of phylum Desulfobacterota has a high abundance of thiosulfate reductase (EC: 1.8.5.5). The BS14_bin17 and SR1_bin13 belonging to the phylum Proteobacteria have a high abundance of sulfur-oxidizing enzymes (Figure 12C). The MAGs of BS14 have a high abundance of enzymes responsible for methanogenesis as compared with SR1 (Figure 12D). The MAGs were also compared for the presence of the enzymes involved in hydrocarbon biodegradation. The MAGs of BS14 were found more enriched with hydrocarbon degradation than SR1 (Supplementary Figures S8A,B). The protocatechuate and benzene modules were found predominant in both metagenomes. For SR1, metagenome SR1_bin2 and SR1_bin8 have the maximum number of genes responsible for benzene degradation, whereas for BS14, BS14_bin7, BS14_bin17, and BS14_bin22 have the highest number of genes responsible for hydrocarbon degradation.

Comparing Electron Transport Chain (ETC) complexes, an almost similar distribution pattern in the MAGs of both SR1 and BS14 was observed, but the MAGs obtained from BS14 also have a high abundance of cytochrome c oxidase, cbb3 type among them (Supplementary Figures S7A,B). At the same time, the presence of arsenate reductase was dominant in the MAGs of BS14 compared with SR1. The photosynthesis module was not observed in any MAG, whereas the module of methanogenesis and methanotrophy, short-chain fatty acid (SCFA), and alcohol conversions were observed in both BS14 and SR1.

The annotation of MAGs of both SR1 and BS14 indicated the microbiome's diverse functional metabolic potential that balances the community's geochemical and energy flux.

## Conclusion

The CETP, Baddi, receives wastewater from hundreds of small and large-scale industries, thus providing an artificial enriched medium to encourage the metabolism of its native microbial community. This study involved high-throughput metagenomic sequencing of activated sludge collected from CETP and river water from their drainage point to decipher the structure of microbial communities and their metabolic potential. The AMR profile and their origin were also determined to study the shared resistome of activated sludge and river water. Our study concluded the prevalence of the bacterial kingdom in activated sludge with the dominance of proteobacteria. On the contrary, the eukarya were dominant in the SR1, with Ciliophora the most abundant phyla. The shared resistome profile was observed in comparing the AMR profile of BS14 and SR1. AMR against cephalosporin, penam, fluoroquinolones, macrolides, and tetracycline was highest in BS14 and SR1. *Azoarcus* and *Desulfococcus* possessed the highest antimicrobial resistance genes against cephalosporins and penams in BS14, whereas, in the case of SR1, *Pseudomonas, Acinetobacter*, and *Shewanella* contained the highest AMR genes for beta-lactam antibiotics. To further explore the spread of disease-causing bacteria and the presence of probable toxins in the SR1 which may originate from BS14, the VFDB analysis revealed that the majority of virulent factors originated from *Desulfuromonas* (6.81%), *Thauera* (6.38%), *Azoarcus* (5.35%), *Acinetobacter* (4.48%), *Geoalkalibacter* (3.25%), *Desulfobulbus* (1.86%) in BS14, and *Pseudomonas* (30.91%) contributed to more virulent factors followed by *Shewanella* (9.71%), *Acinetobacter* (9.28%), *Polynucleobacter* (7.36), *Flavobacteriia* (3.88%), and *Fluviibacter* (3.37%) in SR1. The toxins, i.e., Hemolysin A&B and cyclolysin secretion protein (Cya), were found in both the BS14 and SR1 samples. However, it can be assumed that cyclolysin may also be present in both samples. Colibactin (clbI; a genotoxic metabolite) was found in SR1 but its resistance mechanism was found in BS14. The CAZy annotation showed that the carbohydrate-active enzymes belonging to class GH and GT were most prevalent in both BS14 and SR1. The top carbohydrate-active enzymes obtained from the KEGG database showed the abundance of lipid metabolism, citric acid intermediary enzymes, glycogen synthesis, and enzymes of other central metabolic pathways. On analysis of BS14 and SR1 with PULDB, it was observed that the major community belonged to Proteobacteria and Bacteroidetes for the metabolism of capsular polysaccharides, xylan, lichenan, beta-glucan, etc., mainly in identifying the capability to utilize the 2nd generation feedstock. This study also reported various unique KEGG orthology terms related to different functional categories, such as the biodegradation of xenobiotics and aromatics. The COG and PFAM annotation of the BS14 and SR1 samples depicted the abundance of lipid metabolism, signal transduction mechanisms, membrane integrity, carbohydrate metabolism, and other essential cell regulatory processes. During the functional annotation of MAGs, the pathways related to rTCA and chemolithotropism were observed, which proved the diversity in energy flux and biogeochemical cycles operated in the microbial community. More research is clearly needed to better understand the fate of these genetic components throughout WWTPs to avoid their release from WWTP effluents and reuse of treated water.

## Data Availability Statement

The datasets presented in this study can be found in online repositories. The names of the repository/repositories and accession number(s) can be found in the article/Supplementary Material.

## Author Contributions

GV, HS, and SP designed the experiments, analyzed the data, and wrote the manuscript. AP supervised the project and reviewed the manuscript. All authors contributed to the article and approved the submitted version.

## Funding

This study was supported by the Council of Scientific and Industrial Research (CSIR), India BSC-0402 (CSIR), OLP-804 (CSIR), and the Department of Biotechnology, Government of India (BT/PR7368/INF/22/177/2012).

## Conflict of Interest

The authors declare that the research was conducted in the absence of any commercial or financial relationships that could be construed as a potential conflict of interest.

## Publisher's Note

All claims expressed in this article are solely those of the authors and do not necessarily represent those of their affiliated organizations, or those of the publisher, the editors and the reviewers. Any product that may be evaluated in this article, or claim that may be made by its manufacturer, is not guaranteed or endorsed by the publisher.
