# Supplementary material for: Metagenomics: An Approach for Unraveling the Community Structure and Functional Potential of Activated Sludge of a Common Effluent Treatment Plant
Source: Front Microbiol. 2022 Jul 18;13:933373. doi: 10.3389/fmicb.2022.933373 (PMC9358654; doi:10.3389/fmicb.2022.933373)
Supplement: Supplementary file 1 [file Table_1.DOCX]

**Supplementary Information**

Metagenomics Approach for Unraveling the Community Structure and Functional Potential of Activated Sludge of a Common Effluent Treatment Plant

Authors; Gunjan Vasudeva^†^, Harpreet Singh^†^, Sakshi Paliwal, Anil Kumar Pinnaka*

MTCC-Microbial Type Culture Collection & Gene Bank, CSIR-Institute of Microbial Technology, Chandigarh-160036, India.

^†^Authors contributed equally

*Corresponding Authors: *Dr. Anil Kumar Pinnaka

E-mail: apinnaka@imtech.res.in

Telephone: +91-172-2880728

**Fig. S1** The KEGG distribution profile of BS14 and SR1 shows the abundance of different pathways.

**Fig. S2** The Functional categories predicted by KEGG analysis.

**Fig. S3** The number of ORFs mapped against xenobiotic degradation and metabolism for BS14 and SR1.

**Fig. S4** Metagenomic ORFs mapped to beta-lactam resistance genes.

**Fig. S5** Heatmap showing the PFAM functional assignment of BS14 and SR1 metagenome.

**Fig. S6** Heatmap showing the top 50 KEGG assigned carbohydrate-active enzymes of: **(A)** BS14 and; **(B)** SR1.

**Fig. S7** Functional annotation of MAGs obtained by DRAM: **(A)** BS14 and; **(B)** SR1.

**Fig. S8** Functional annotation of MAGs for the presence of crucial enzymes involved in hydrocarbon biodegradation: **(A)** BS14 and; **(B)** SR1.

**Table S1.** Genomic feature summary of MAGs in SR1 metagenome using checkm. The marker lineage is expressed at different phylogenetic clades, k; kingdom, p; phylum, c; class, o; order, f; family, g; genus, s; species.

**Table S2.** Genomic feature summary of MAGs in BS14 metagenome using checkm. The marker lineage is expressed at different phylogenetic clades, k; kingdom, p; phylum, c; class, o; order, f; family, g; genus, s; species.

**Table S3.** Taxonomic assignment and abundance of MAGs in SR1.

**Table S4.** The taxonomic assignment and abundance of MAGs in BS14.

**Fig. S1**


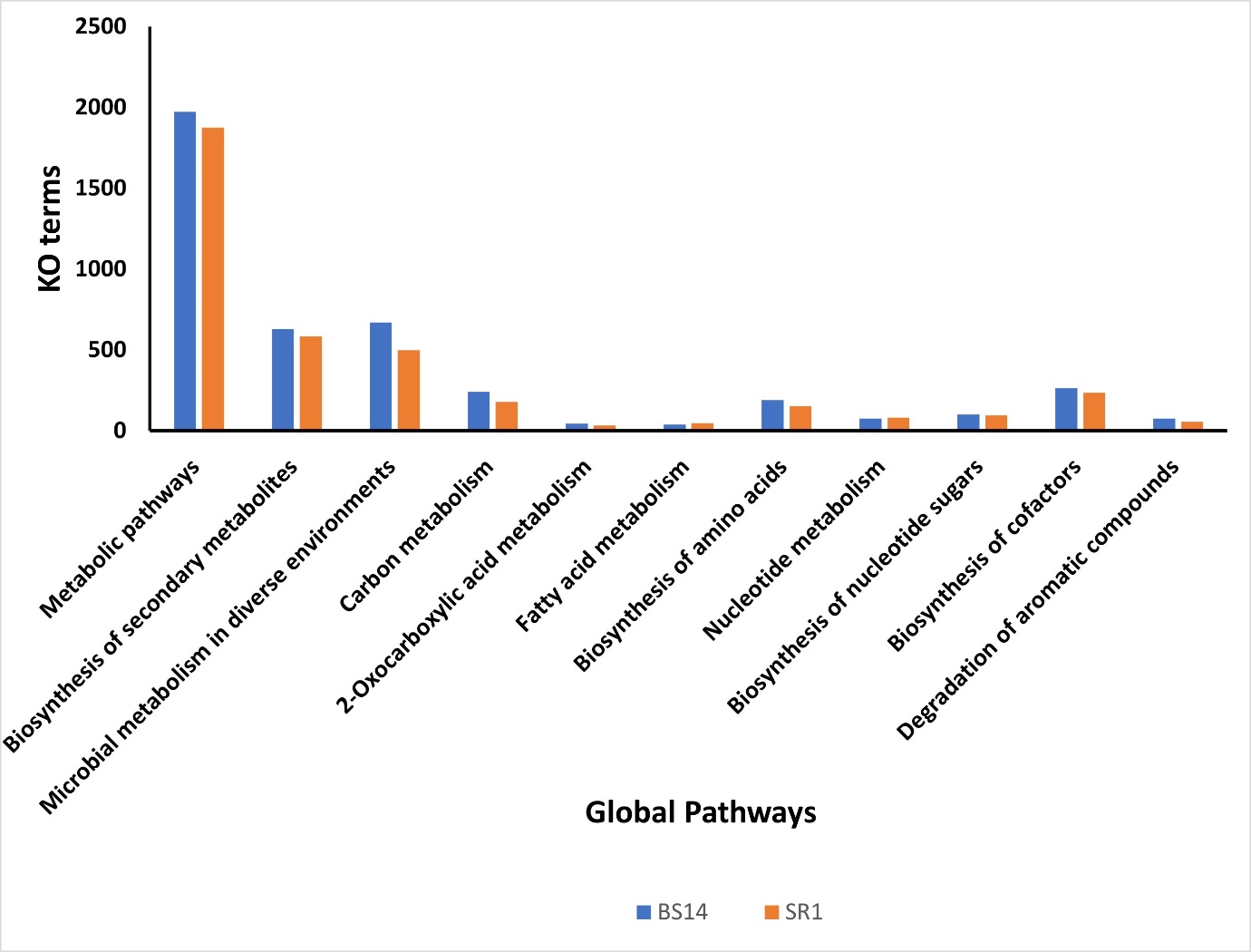


**Fig. S2**


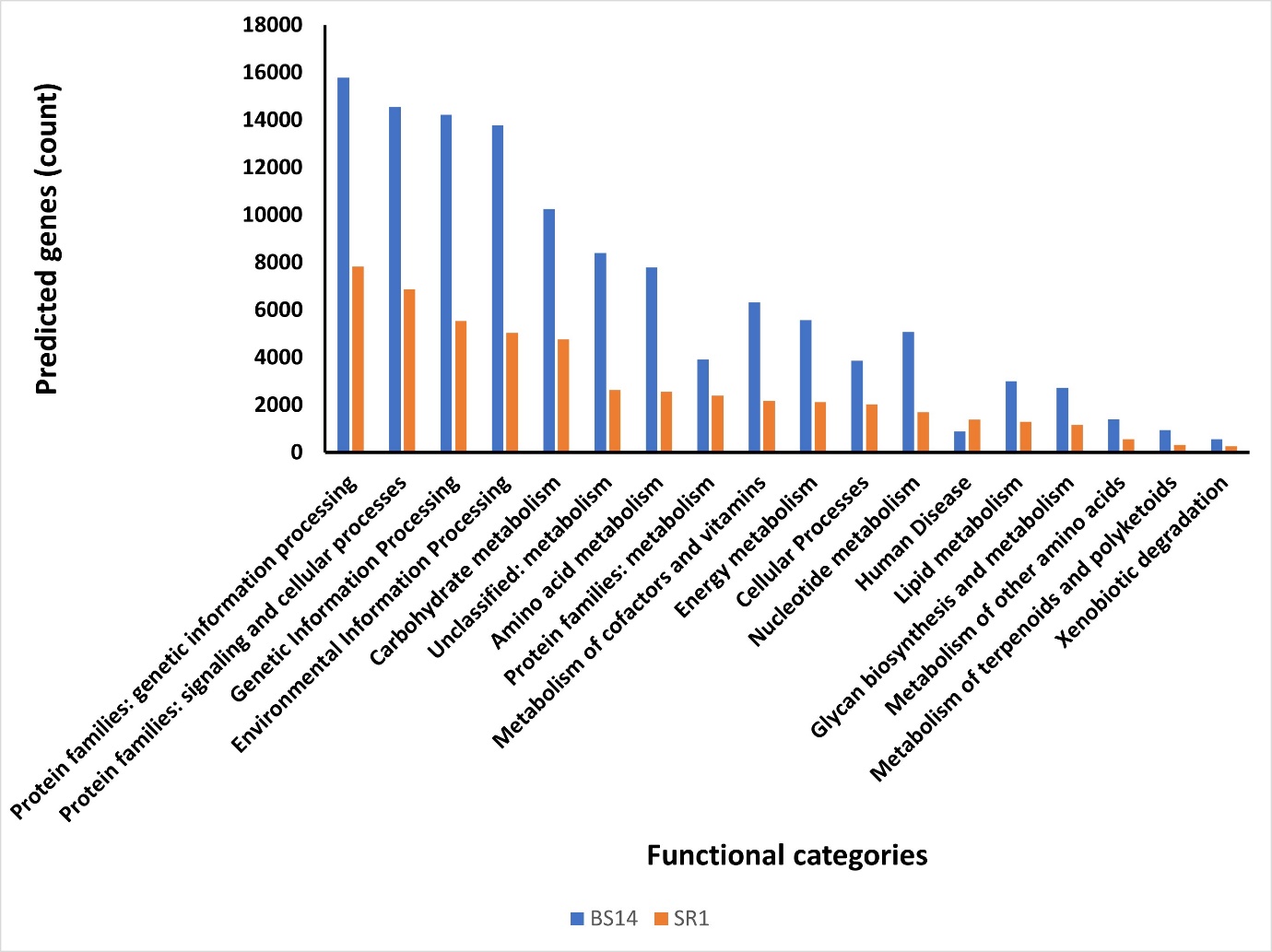


**Fig. S3**


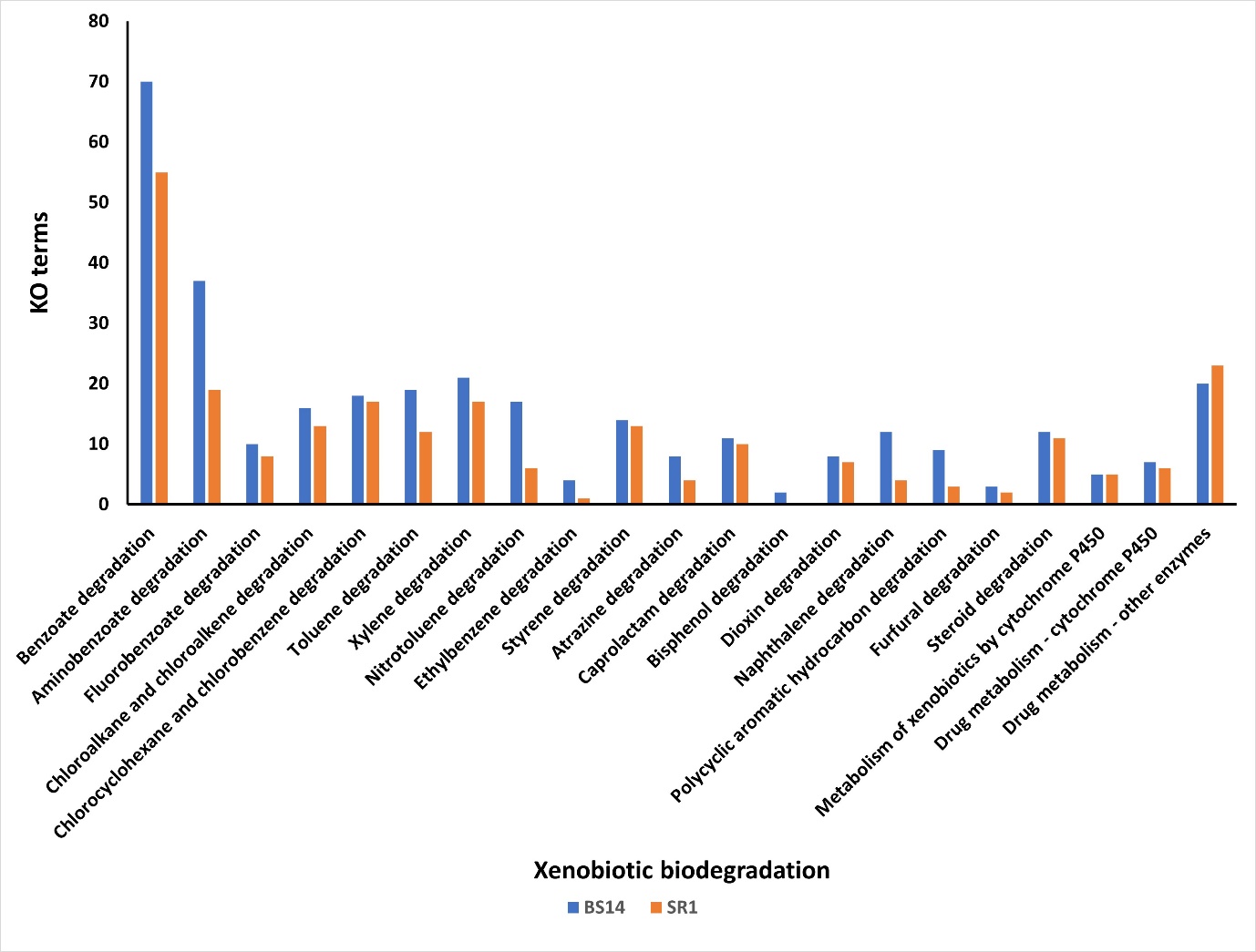


**
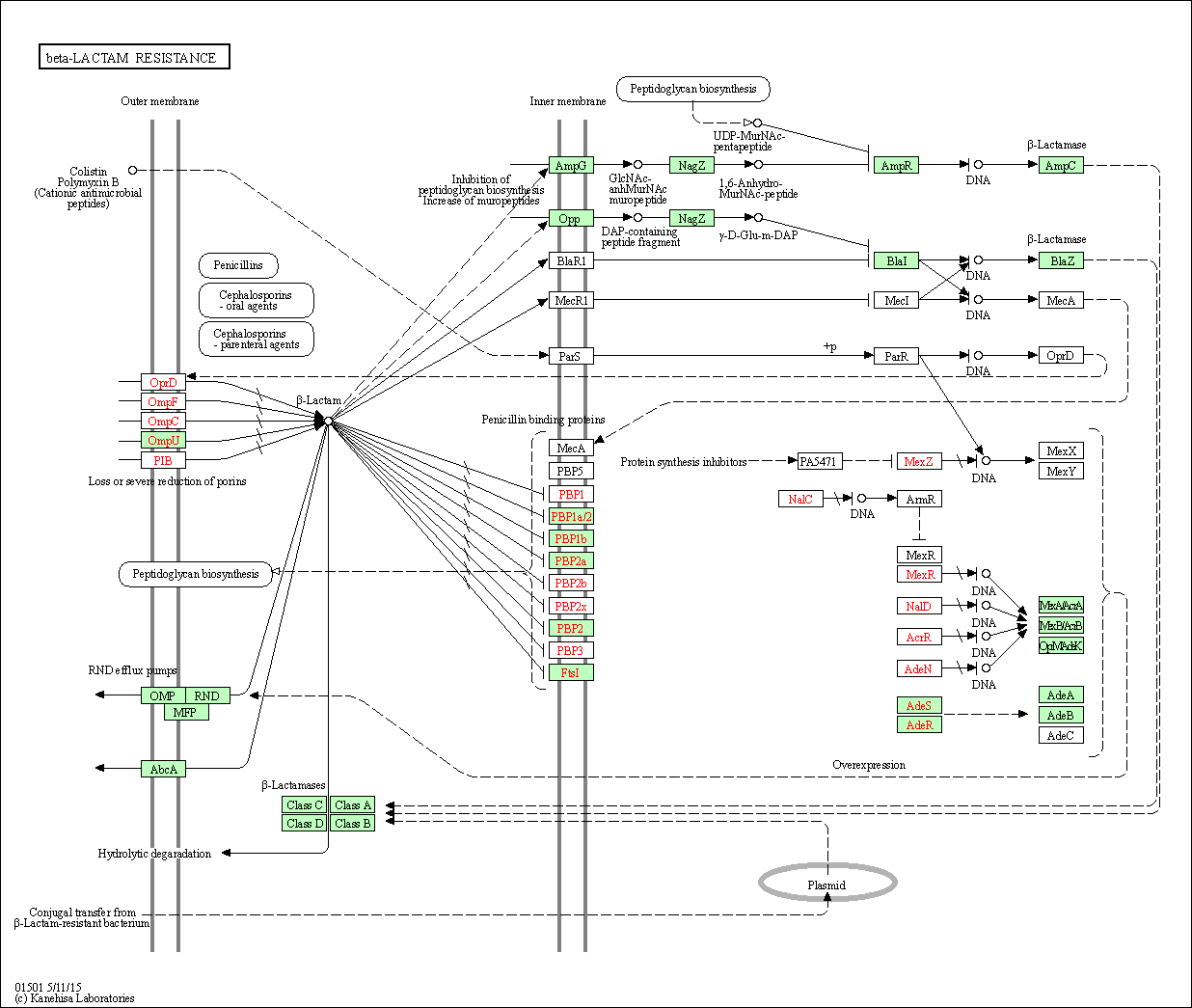
Fig. S4**

**Fig. S5**


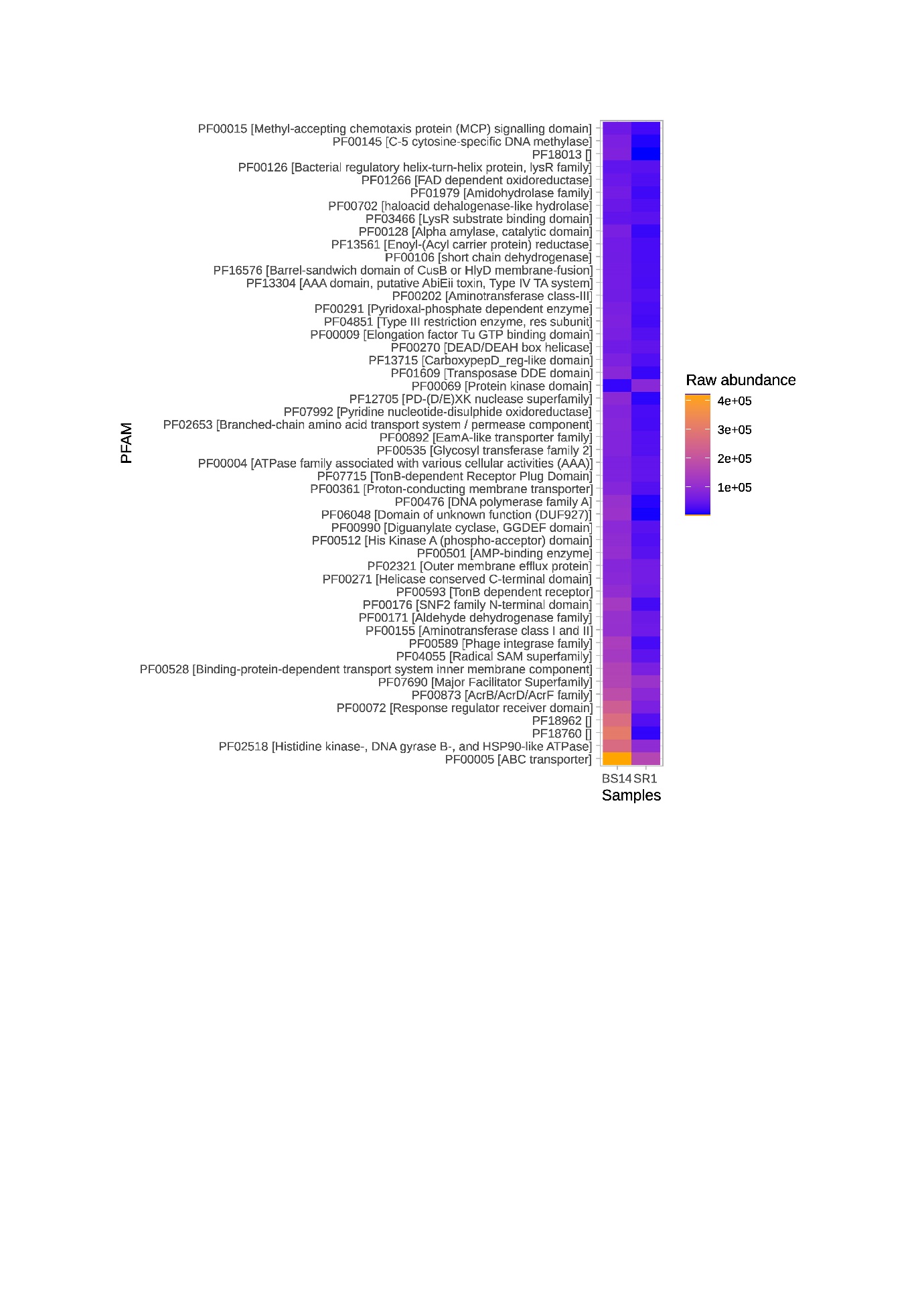


**Fig. S6A**


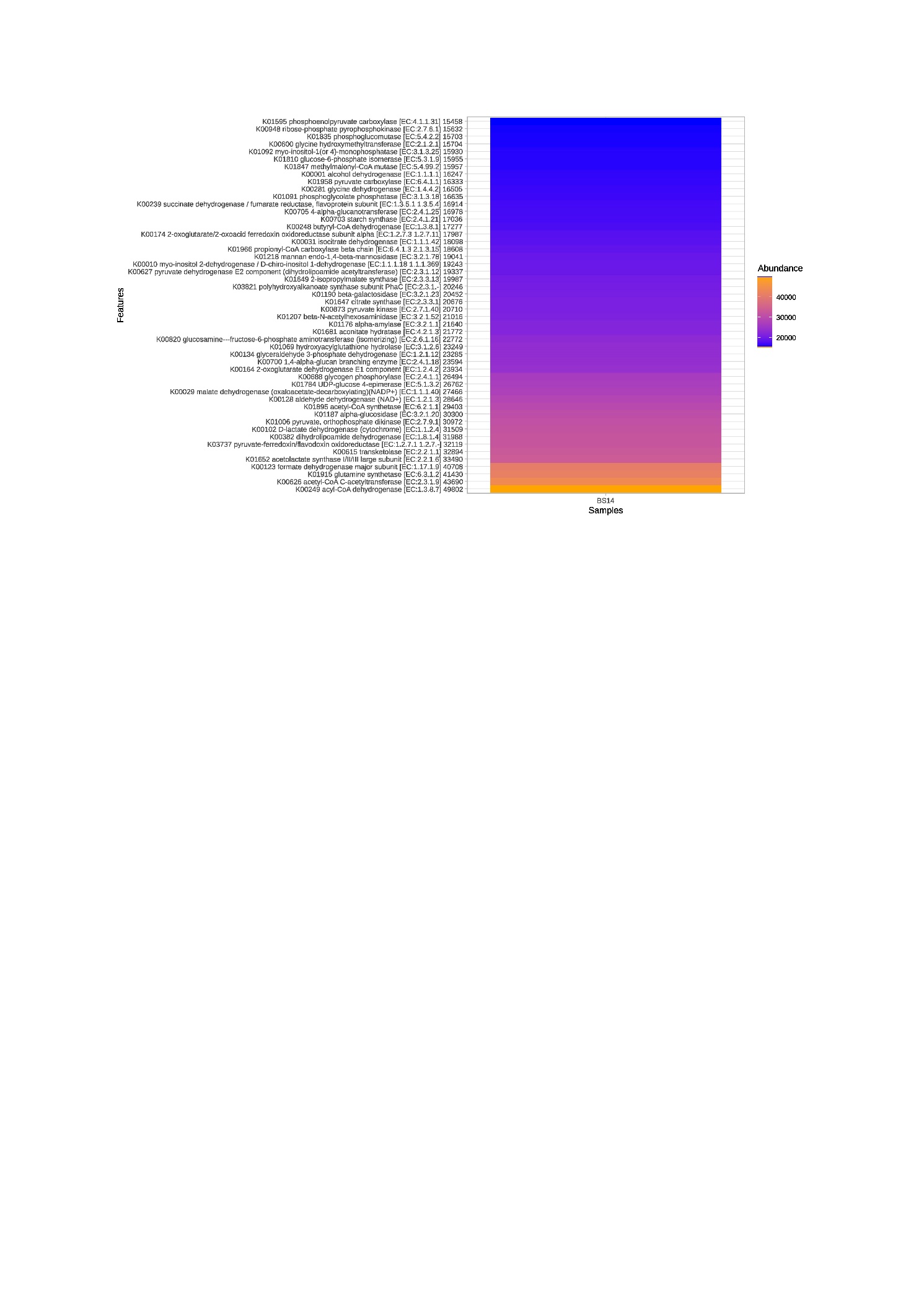


**
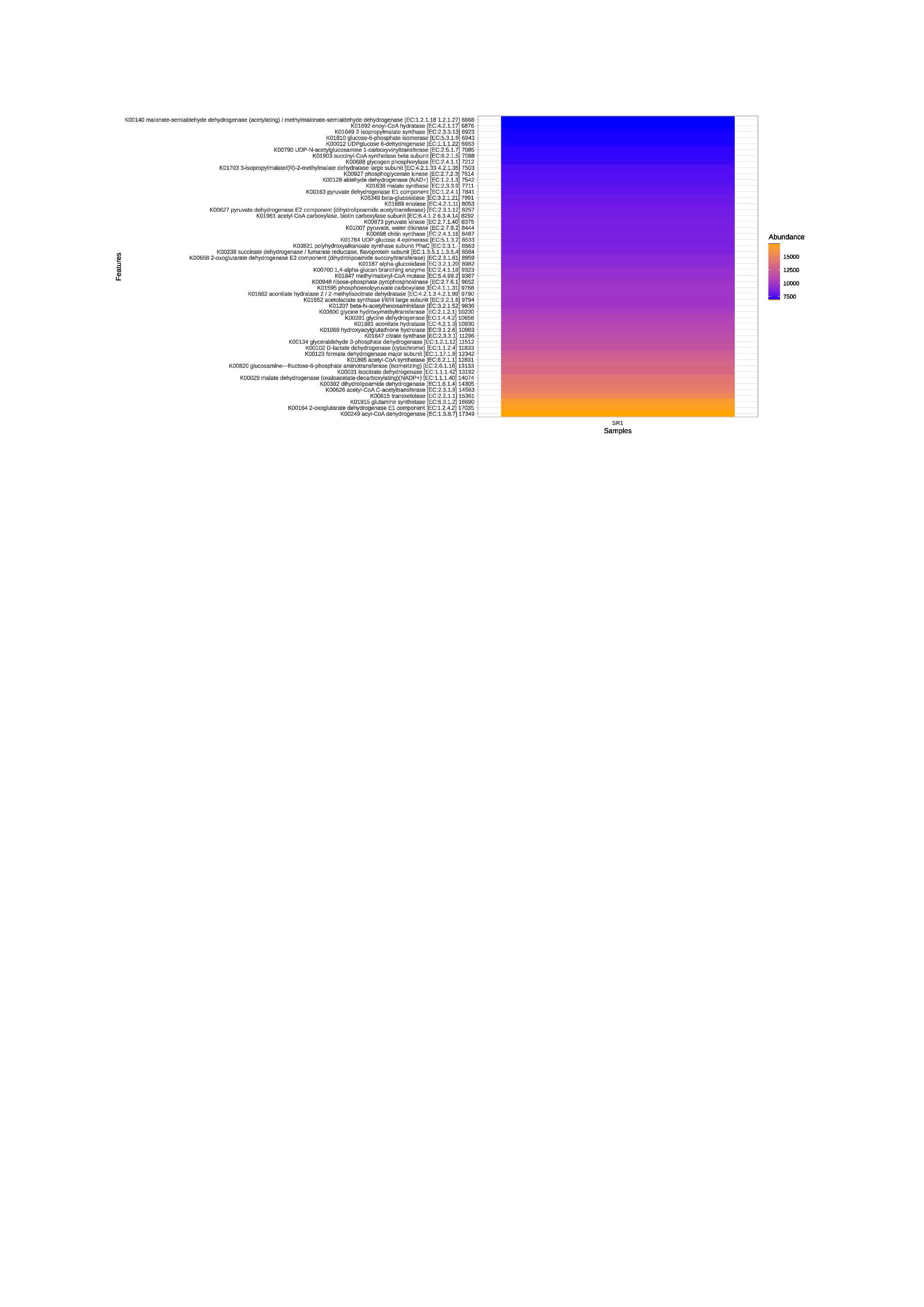
Fig. S6B**

**Fig. S7A**


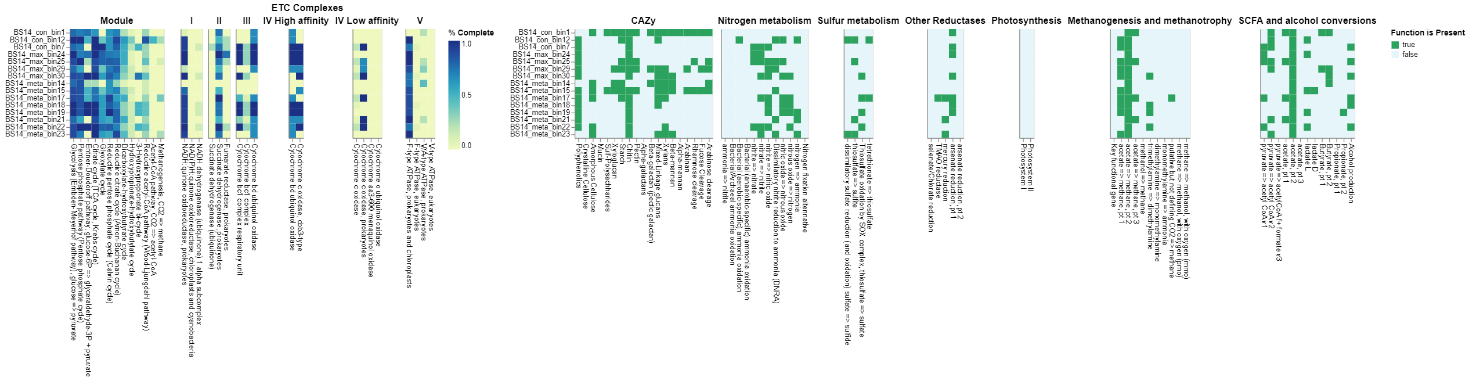


**Fig. S7B**


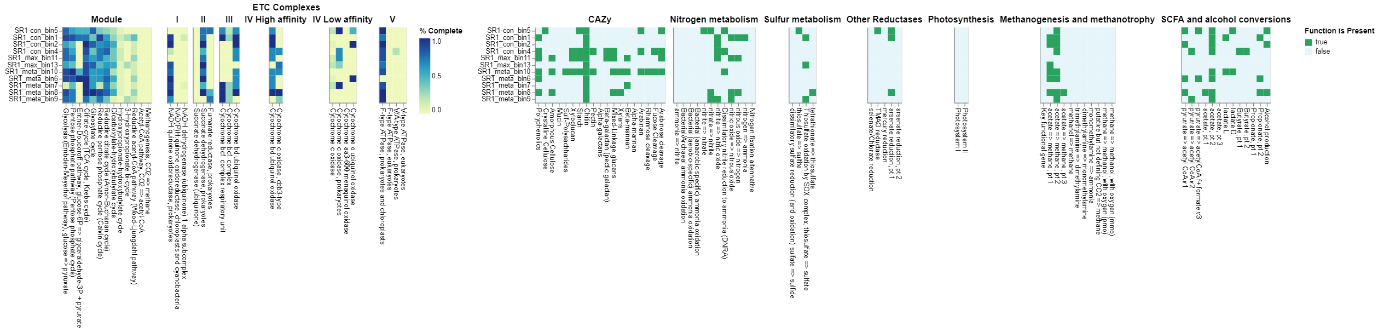


**Fig. S8A**


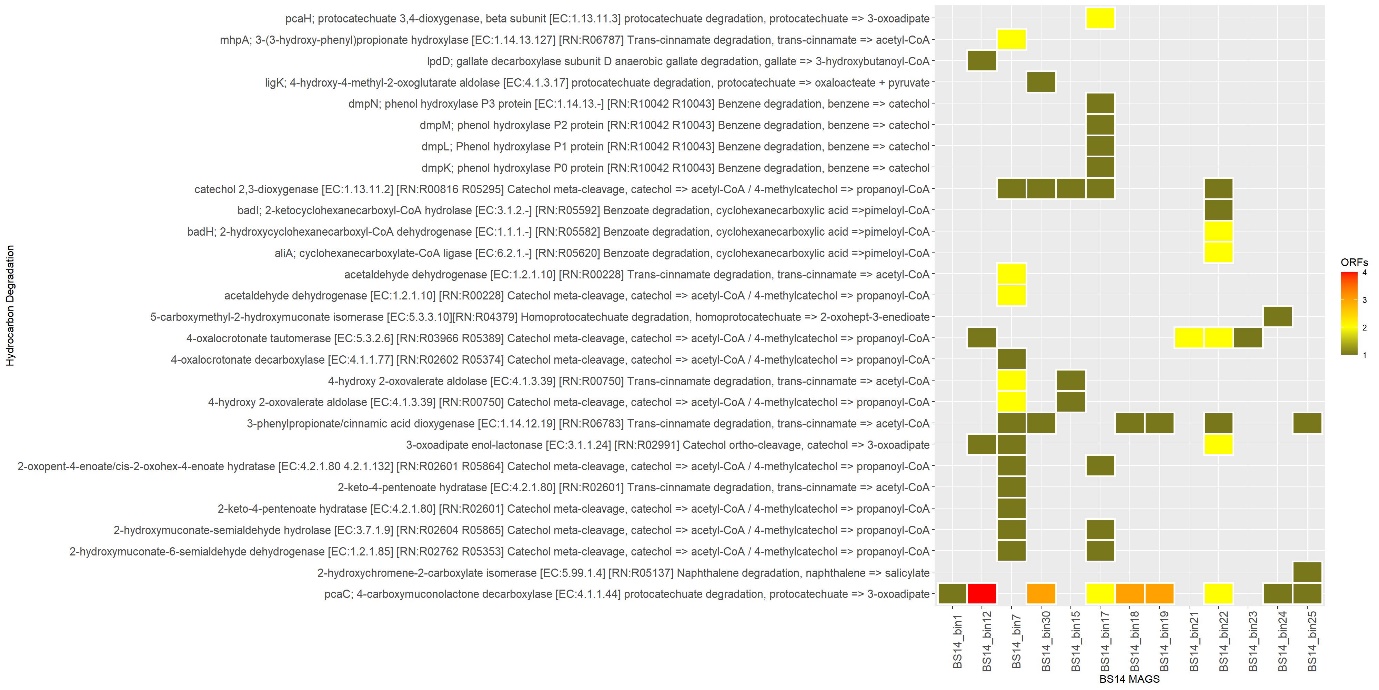


**Fig. S8B**


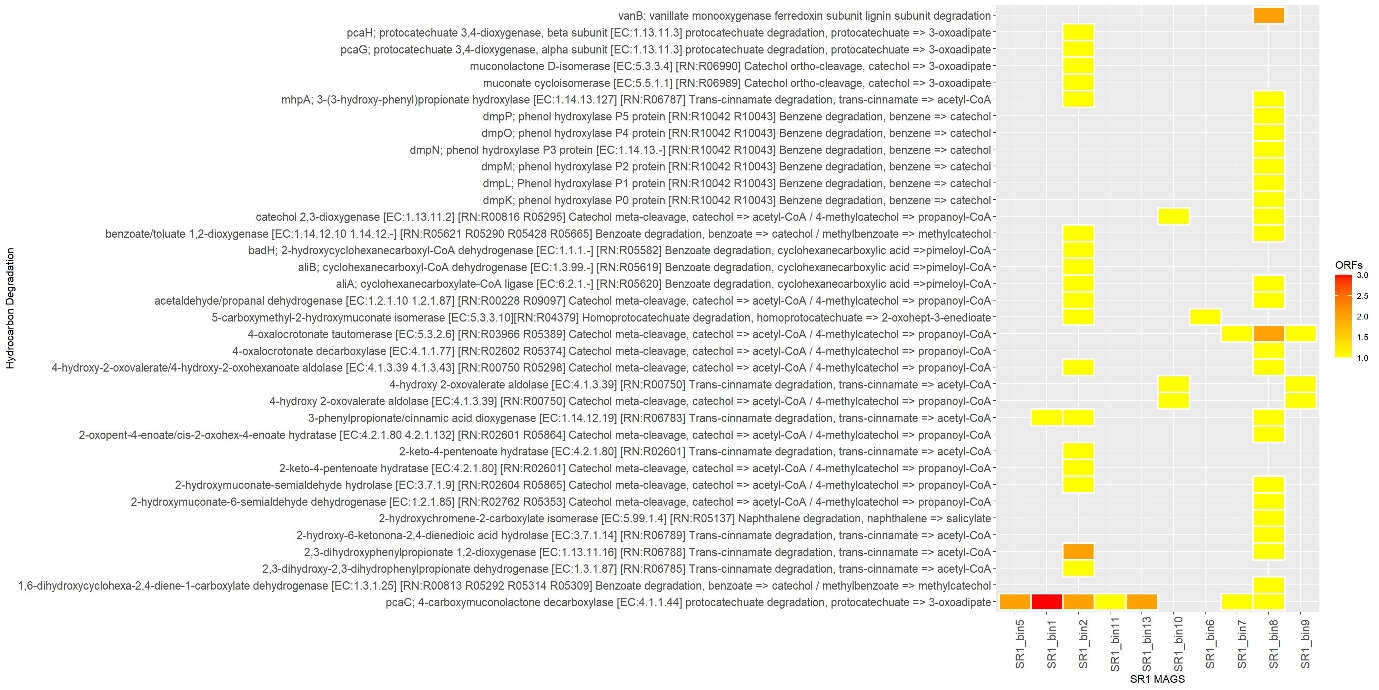


**Table S1.** Genomic feature summary of MAGs in SR1 metagenome using checkm. The marker lineage is expressed at different phylogenetic clades, k; kingdom, p; phylum, c; class, o; order, f; family, g; genus, s; species.

| Bin Id | Marker lineage | Genomes | Markers | Completeness | Contamination | Strain heterogeneity |
| --- | --- | --- | --- | --- | --- | --- |
| SR1_bin8 | f__Rhodocyclaceae (UID3972) | 30 | 241 | 99.17 | 0.76 | 0.00 |
| SR1_bin10 | k__Bacteria (UID2982) | 88 | 230 | 98.99 | 2.93 | 0.00 |
| SR1_bin7 | o__Rickettsiales | 83 | 324 | 97.63 | 97.63 | 97.63 |
| SR1_bin15 | c__Betaproteobacteria (UID3971) | 223 | 425 | 96.42 | 113.84 | 35.03 |
| SR1_bin14 | f__Flavobacteriaceae (UID2817) | 81 | 511 | 94.64 | 19.47 | 16.09 |
| SR1_bin3 | o__Burkholderiales (UID4001) | 108 | 570 | 94.51 | 17.24 | 47.41 |
| SR1_bin6 | p__Proteobacteria (UID3880) | 1495 | 261 | 93.22 | 3.35 | 16.67 |
| SR1_bin1 | f__Rhodobacteraceae (UID3340) | 84 | 568 | 91.65 | 0.96 | 50.00 |
| SR1_bin9 | k__Bacteria (UID3060) | 138 | 338 | 89.84 | 0.41 | 100.00 |
| SR1_bin4 | k__Bacteria (UID2569) | 434 | 278 | 88.33 | 3.76 | 12.50 |
| SR1_bin2 | f__Moraxellaceae (UID4680) | 86 | 689 | 87.17 | 0.82 | 50.00 |
| SR1_bin5 | p__Proteobacteria (UID3880) | 1495 | 261 | 84.59 | 6.10 | 7.69 |
| SR1_bin13 | o__Burkholderiales (UID4001) | 108 | 570 | 80.42 | 1.90 | 50.00 |
| SR1_bin11 | f__Flavobacteriaceae (UID2817) | 81 | 511 | 80.11 | 2.60 | 0.00 |
| SR1_bin12 | k__Archaea (UID2) | 207 | 149 | 74.93 | 30.12 | 2.83 |
| SR1-bin5 | o__Alteromonadales (UID4837) | 30 | 868 | 73.77 | 7.33 | 76.67 |
| SR1_bin16 | c__Gammaproteobacteria (UID4761) | 52 | 693 | 16.31 | 1.44 | 6.67 |

**Table S2.** Genomic feature summary of MAGs in BS14 metagenome using checkm. The marker lineage is expressed at different phylogenetic clades, k; kingdom, p; phylum, c; class, o; order, f; family, g; genus, s; species.

| Bin Id | Marker lineage | Genomes | Markers | Completeness | Contamination | Strain heterogeneity |
| --- | --- | --- | --- | --- | --- | --- |
| BS14_bin24 | c__Betaproteobacteria (UID3888) | 323 | 387 | 98.58 | 0.32 | 0.00 |
| BS14_bin1 | k__Bacteria (UID2570) | 433 | 273 | 98.36 | 1.82 | 75.00 |
| BS14_bin18 | f__Rhodobacteraceae (UID3340) | 84 | 568 | 98.28 | 0.12 | 0.00 |
| BS14_bin19 | o__Rhizobiales (UID3450) | 238 | 513 | 97.99 | 0.86 | 14.29 |
| BS14_bin22 | c__Gammaproteobacteria (UID4202) | 67 | 481 | 97.66 | 4.09 | 7.69 |
| BS1_bin25 | c__Deltaproteobacteria (UID3217) | 62 | 280 | 97.38 | 2.38 | 0.00 |
| BS14_bin23 | c__Deltaproteobacteria (UID3216) | 83 | 247 | 96.59 | 1.67 | 0.00 |
| BS14_bin12 | f__Rhodobacteraceae (UID3340) | 84 | 568 | 96.11 | 0.00 | 0.00 |
| BS14_bin30 | k__Bacteria (UID2569) | 434 | 278 | 95.97 | 1.88 | 0.00 |
| BS14_bin29 | c__Alphaproteobacteria (UID3305) | 564 | 349 | 95.87 | 0.00 | 0.00 |
| BS14_bin7 | k__Bacteria (UID2982) | 88 | 230 | 93.54 | 2.93 | 16.67 |
| BS14_bin15 | c__Deltaproteobacteria (UID3216) | 83 | 247 | 92.90 | 0.00 | 0.00 |
| BS14_bin21 | f__Rhodocyclaceae (UID3972) | 30 | 540 | 92.15 | 3.62 | 11.54 |
| BS14_bin17 | k__Bacteria (UID2328) | 3167 | 126 | 92.00 | 0.00 | 0.00 |
| BS14_bin14 | k__Bacteria (UID1452) | 924 | 163 | 89.44 | 4.83 | 50.00 |
| BS14_bin4 | f__Moraxellaceae (UID4680) | 86 | 689 | 88.99 | 4.17 | 72.00 |
| BS14_bin8 | k__Bacteria (UID2329) | 174 | 149 | 87.93 | 18.93 | 10.71 |
| BS14_bin2 | k__Bacteria (UID2982) | 88 | 230 | 87.12 | 3.41 | 12.50 |
| BS14_bin16 | c__Gammaproteobacteria (UID4274) | 112 | 581 | 84.02 | 0.95 | 100.00 |
| BS14_bin20 | o__Clostridiales (UID1212) | 172 | 263 | 83.35 | 2.01 | 75.00 |
| BS14_bin13 | k__Bacteria  (UID2569) | 434 | 278 | 82.65 | 3.32 | 22.22 |
| BS14_bin3 | k__Bacteria (UID2329) | 174 | 149 | 80.94 | 3.93 | 25.00 |
| BS14_bin11 | k__Bacteria (UID1452) | 924 | 163 | 80.45 | 6.19 | 20.00 |
| BS14_bin26 | k__Bacteria (UID2982) | 88 | 230 | 78.60 | 60.65 | 68.80 |
| BS14_bin27 | k__Bacteria (UID2495) | 2993 | 142 | 75.11 | 0.98 | 60.00 |
| BS14_bin5 | k__Bacteria (UID209) | 5443 | 105 | 68.75 | 5.24 | 50.00 |
| BS14_bin6 | k__Bacteria (UID203) | 5449 | 104 | 68.23 | 33.08 | 0 |
| BS14_bin28 | k__Bacteria (UID2328) | 3167 | 126 | 58.52 | 0.12 | 0 |
| BS14_bin9 | o__Clostridiales (UID1120) | 304 | 250 | 45.41 | 3.51 | 11.11 |

**Table S3.** Taxonomic assignment and abundance of MAGs in SR1.

| **Bin Id** | **Phylum** | **Class** | **order** | **Family** | **Genus** | **Species** | **Abundance** | **Relative Abundance** |
| --- | --- | --- | --- | --- | --- | --- | --- | --- |
| SR1 _bin1 | Proteobacteria | Alphaproteobacteria | Rhodobacterales | Rhodobacteraceae | Rhodobacter | -- | 0.020 | 4.915985037 |
| SR1_bin5 | Proteobacteria | Gammaproteobacteria | Enterobacterales | Shewanellaceae | Shewanella | -- | 0.19 | 4.63 |
| SR1 _bin2 | Proteobacteria | Gammaproteobacteria | Pseudomonadales | Moraxellaceae | Acinetobacter | bohemicus | 0.064 | 15.99089776 |
| SR1 _bin4 | Bacteroidota | Bacteroidia | Bacteroidales | 4484-276 | RZYY01 | -- | 0.010 | 2.571795511 |
| SR1_ bin11 | Bacteroidota | Bacteroidia | Flavobacteriales | Flavobacteriaceae | Flavobacterium | -- | 0.089 | 22.10027431 |
| SR1_ _bin13 | Proteobacteria | Gammaproteobacteria | Burkholderiales | Burkholderiaceae | Polynucleobacter | yangtzensis | 0.085 | 21.18206983 |
| SR1_ bin10 | Verrucomicrobiota | Verrucomicrobiae | Chthoniobacterales | JACTMZ01 | JACTMZ01 | -- | 0.016 | 3.884812968 |
| SR1_bin6 | Proteobacteria | Gammaproteobacteria | Burkholderiales | UBA11063 | UBA11063 | -- | 0.022 | 5.599551122 |
| SR1_bin7 | Proteobacteria | Alphaproteobacteria | Rickettsiales | Rickettsiaceae | GCA-2402195 | -- | 0.028 | 6.950498753 |
| SR1_bin8 | Proteobacteria | Gammaproteobacteria | Burkholderiales | Rhodocyclaceae | Thauera | -- | 0.040 | 9.901421446 |
| SR1_bin9 | Campylobacterota | Campylobacteria | Campylobacterales | Arcobacteraceae | Aliarcobacter | cryaerophilus_A | 0.028 | 6.903640898 |
| SR1_bin1 | Proteobacteria | Gammaproteobacteria | Burkholderiales | Burkholderiaceae | Polynucleobacter | yangtzensis | 0.020 | 4.915985037 |
| SR1_bin2 | Verrucomicrobiota | Verrucomicrobiae | Chthoniobacterales | JACTMZ01 | JACTMZ01 | -- | 0.064 | 15.99089776 |

**Table S4.** The taxonomic assignment and abundance of MAGs in BS14.

| **Bin Id** | **Phylum** | **Class** | **order** | **Family** | **Genus** | **Species** | **Abundance** | **Relative Abundance** |
| --- | --- | --- | --- | --- | --- | --- | --- | --- |
| BS14_bin1 | Bacteroidota | Bacteroidia | Bacteroidales | Dysgonomonadaceae | UBA4179 | UBA4179 sp002849245 | 0.007 | 1.695094 |
| BS14_bin12 | Desulfobacterota | Syntrophobacteria | Syntrophobacterales | Syntrophobacteraceae | DSUX01 | -- | 0.005 | 1.335512 |
| BS14_bin7 | Proteobacteria | Alphaproteobacteria | Sphingomonadales | Sphingomonadaceae | -- | -- | 0.021 | 5.270616 |
| BS14_bin29 | Bacteroidota | Bacteroidia | Bacteroidales | 4484-276 | RZYY01 | -- | 0.051 | 12.51707 |
| BS14_bin30 | Proteobacteria | Alphaproteobacteria | Rhodobacterales | Rhodobacteraceae | -- | -- | 0.049 | 12.0947 |
| BS14_bin14 | Firmicutes | Bacilli | Izemoplasmatales | UBA5603 | UBA5603 | -- | 0.005 | 1.197047 |
| BS14_bin15 | Verrucomicrobiota | Verrucomicrobiae | Chthoniobacterales | JACTMZ01 | JACTMZ01 | -- | 0.007 | 1.71965 |
| BS14_bin17 | Proteobacteria | Gammaproteobacteria | Burkholderiales | Rhodocyclaceae | Thauera | Thauera propionica | 0.020 | 4.938744 |
| BS14_bin18 | Proteobacteria | Alphaproteobacteria | Rhodobacterales | Rhodobacteraceae | LPB0142 | LPB0142 sp001856665 | 0.040 | 9.960887 |
| BS14_bin19 | Proteobacteria | Alphaproteobacteria | Rhodobacterales | Rhodobacteraceae | Rhodobacter | -- | 0.069 | 16.9252 |
| BS14_bin21 | Desulfobacterota_ | Desulfuromonadia | Desulfuromonadales | Geoalkalibacteraceae | Geoalkalibacter | -- | 0.024 | 5.832291 |
| BS14_bin22 | Proteobacteria | Alphaproteobacteria | Rhizobiales | Rhizobiaceae | Aquamicrobium_A | -- | 0.021 | 5.198818 |
| BS14_bin23 | Desulfobacterota | Desulfobulbia | Desulfobulbales | Desulfobulbaceae | Desulfobulbus | Desulfobulbus propionicus | 0.010 | 2.371333 |
| BS14_bin24 | Proteobacteria | Gammaproteobacteria | Burkholderiales | Burkholderiaceae | -- | -- | 0.043 | 10.57143 |
| BS14_bin25 | Proteobacteria | Gammaproteobacteria | Steroidobacterales | Steroidobacteraceae | RPQJ01 | -- | 0.034 | 8.48069 |
